# The Usher syndrome 1C protein harmonin regulates canonical Wnt signaling

**DOI:** 10.3389/fcell.2023.1130058

**Published:** 2023-02-08

**Authors:** Jessica Schäfer, Nicole Wenck, Katharina Janik, Joshua Linnert, Katarina Stingl, Susanne Kohl, Kerstin Nagel-Wolfrum, Uwe Wolfrum

**Affiliations:** ^1^ Institute of Molecular Physiology, Molecular Cell Biology and Photoreceptor Cell Biology, Johannes Gutenberg University Mainz, Mainz, Germany; ^2^ Centre for Ophthalmology, University Eye Hospital, University of Tübingen, Tübingen, Germany; ^3^ Centre for Ophthalmology, Institute for Ophthalmic Research, University of Tübingen, Tübingen, Germany; ^4^ Institute of Developmental Biology and Neurobiology, Johannes Gutenberg University Mainz, Mainz, Germany

**Keywords:** Usher syndrome, *USH1C*, *β*-catenin, translational read-through inducing drugs, ataluren/PTC124, colorectal cancer, translational read-through, wnt signaling pathway

## Abstract

Human Usher syndrome (USH) is the most common form of hereditary combined deaf-blindness. USH is a complex genetic disorder, and the pathomechanisms underlying the disease are far from being understood, especially in the eye and retina. The *USH1C* gene encodes the scaffold protein harmonin which organizes protein networks due to binary interactions with other proteins, such as all USH proteins. Interestingly, only the retina and inner ear show a disease-related phenotype, although *USH1C*/harmonin is almost ubiquitously expressed in the human body and upregulated in colorectal cancer. We show that harmonin binds to *β*-catenin, the key effector of the canonical Wnt (cWnt) signaling pathway. We also demonstrate the interaction of the scaffold protein *USH1C*/harmonin with the stabilized acetylated *β*-catenin, especially in nuclei. In HEK293T cells, overexpression of *USH1C*/harmonin significantly reduced cWnt signaling, but a *USH1C*-R31* mutated form did not. Concordantly, we observed an increase in cWnt signaling in dermal fibroblasts derived from an *USH1C* ^R31*/R80Pfs*69^ patient compared with healthy donor cells. RNAseq analysis reveals that both the expression of genes related to the cWnt signaling pathway and cWnt target genes were significantly altered in USH1C patient-derived fibroblasts compared to healthy donor cells. Finally, we show that the altered cWnt signaling was reverted in USH1C patient fibroblast cells by the application of Ataluren, a small molecule suitable to induce translational read-through of nonsense mutations, hereby restoring some USH1C expression. Our results demonstrate a cWnt signaling phenotype in USH establishing *USH1C*/harmonin as a suppressor of the cWnt/*β*-catenin pathway.

## Introduction

The human Usher syndrome (USH) is the most common form of inherited combined deaf-blindness with a prevalence of 1:6,000 to 1:10,000 in human (Kimberling et al., 2010; Friedman et al., 2011). USH is revealed as a ciliopathy presenting defects in ciliary processes (Bujakowska et al., 2017; May-Simera et al., 2017; Samanta et al., 2019). USH disease is clinically and genetically complex and is subdivided into three clinical subtypes (USH 1–3) that are caused by mutations in at least 10 genes (https://databases.lovd.nl/shared/genes/USH1C) (Fuster-Garcia et al., 2021; Delmaghani and El-Amraoui, 2022). USH1 is the most severe type featured by congenital deafness, vestibular function defects, and prepuberty onset of retinitis pigmentosa (RP). So far, six USH1 genes have been identified, namely *MYO7A* (USH1B), *USH1C*, *CDH23* (USH1D), *PCDH15* (USH1F)*, USH1G,* and *CIB2* (USH1J). These USH1 genes encode for proteins of diverse protein families such as motor proteins, transmembrane proteins, and scaffold proteins (Fuster-Garcia et al., 2021; Delmaghani and El-Amraoui, 2022), which are often ubiquitously expressed in the human body, organs, tissues and cells (Reiners et al., 2006).

**FIGURE 1:**
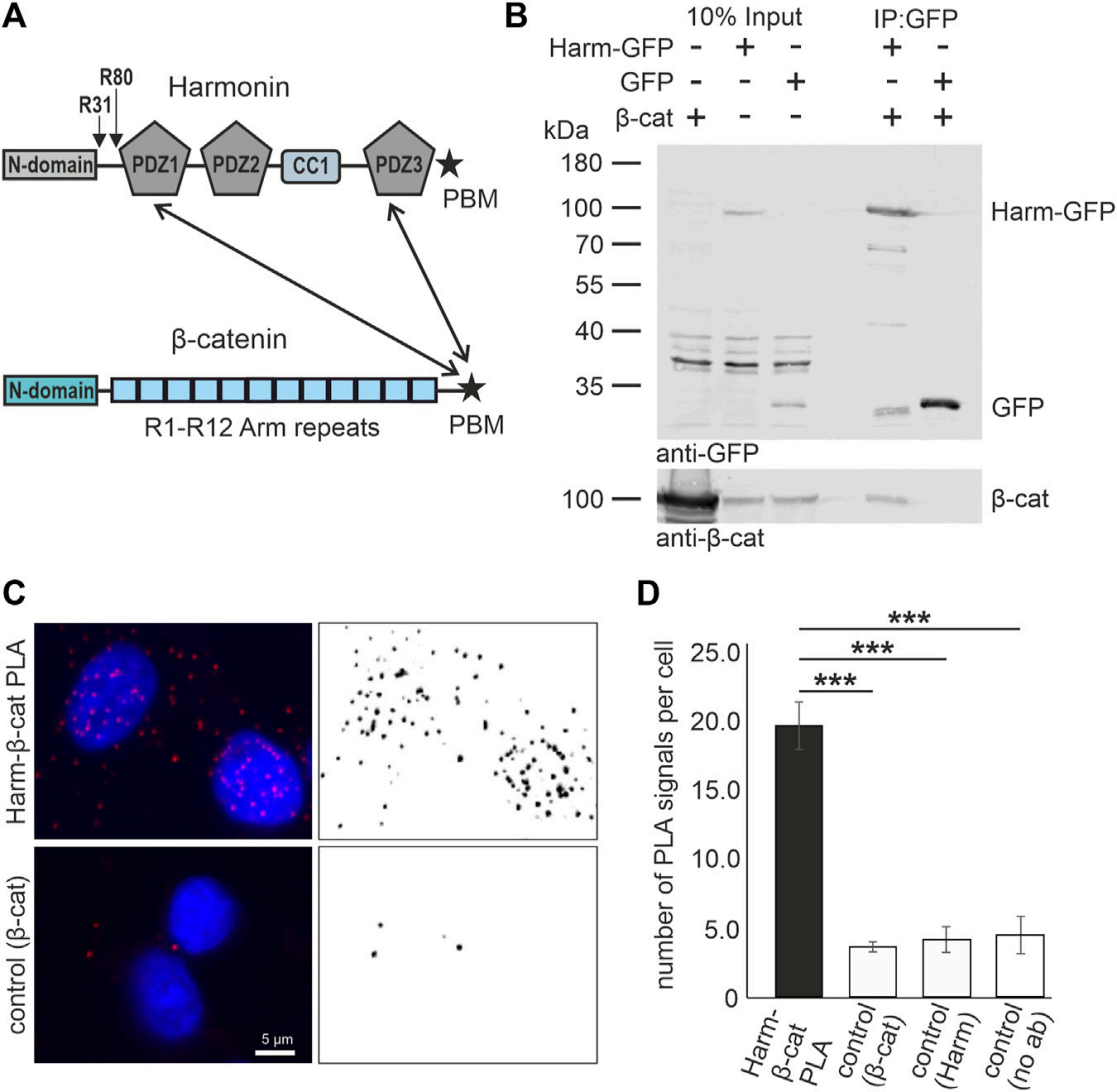
*In vitro* and *in situ* interaction of harmonin with *β*-catenin. **(A)** Schematic representations of the domain structures of the scaffold protein *USH1C*/harmonin (isoform a) and *β*-catenin (*β*-cat). Pathogenic *USH1C* mutations c.91C>T; p.(R31*) and c.238dupC; p.(R80Pfs*69) are highlighted. Twoheaded arrows indicate that the C terminal PDZ (PDZ; PSD-95, DLG, and ZO-1) The pathogenic nonsense mutations c.91C>T; p.(R31*) and c.238dupC; p.(R80Pfs*69) in *USH1C* referred to in the Study are highlighted. Binding motif (PBM) of *β*-catenin is capable to bind to the PDZ1 and PDZ3 of harmonin. **(B)** Western blot analysis of a representative GFP-Trap^®^ demonstrates interaction between harmonin a1-GFP (Harm-GFP) and *β*-catenin (*β*-cat) from lysates of HEK293T cells transfected with *β*-cat and Harm-GFP, or GFP, respectively. Harm-GFP but not GFP alone pulled down *β*-catenin; N = 3 experiments. **(C)** Representative fluorescence image of PLA signals (red, left) in HEK293T cells demonstrates interaction of intrinsic harmonin (Harm) and *β*-cat (upper panel). Signal is significantly decreased in the control using only *β*-cat antibody (lower panel). Cell nuclei are marked with DAPI (blue). **(D)** Statistical analysis reveals an increase in number of signals of 4-fold in the PLA compared to all three controls (Supplementary Figure S1). Two-tailed Student’s *t*-test, ***p ≤ 0.001; N = 3 experiments.

The scaffold protein harmonin is encoded by the *USH1C* gene (ENSG00000006611; OMIM 276904) (Bitner-Glindzicz et al., 2000; Verpy et al., 2000) which consists of 28 exons, and alternative splicing of *USH1C* results in numerous splice variants, which are grouped based on their domain composition into three major splice groups a, b, and c (Nagel-Wolfrum et al., 2022). Although *USH1C*/harmonin is almost ubiquitously expressed in humans, it functions as a key organizer of USH protein networks predominantly in the mechanosensitive hair cells of the inner ear and in retinal cells, namely photoreceptor cells and Müller glia cells, but also in brush border microvilli of intestinal epithelia (Wolfrum, 2011; Crawley et al., 2016; Li et al., 2016; Nagel-Wolfrum et al., 2022). Harmonin molecules can harbor up to three PDZ (named after PSD-95, DLG, and ZO-1) domains (Figure 1A) that are capable of binding of all other known USH proteins, but also other proteins which are often associated with membrane proteins and cytoskeletal proteins, such as JAM-B, rhodopsin or filamin-A (Reiners et al., 2006; Wolfrum, 2011; Nagel-Wolfrum et al., 2022). Recently, we also confirmed the interaction of harmonin with *β*-catenin *via* binding of *β*-catenin´s type-I PDZ binding motif (DTDL = PBM) at the end of the C-terminal domain to the PDZ1 and PDZ3 domains of harmonin (Figure 1A; Nagel-Wolfrum et al., 2022) as previously suggested (Johnston et al., 2004). When *USH1C* is truncated due to the R31* or the R80Pfs*69 (Figure 1A) this particular interaction should be lost.

*β*-catenin is a multitasking and evolutionary conserved protein with dual major functions: it is not only an essential component of cadherin-based cell-cell adhesion complexes in adherent junctions (AJ) but also the key effector of canonical Wnt (cWnt) signaling in the nucleus (Valenta et al., 2012). In the cadherin-catenin core complex of AJs *β*-catenin bridges the p120 catenin, which binds to the cytoplasmic C-terminus of cadherins, to *α*-catenin and thereby to the actin cytoskeleton (Takeichi, 2014). We have recently shown that harmonin and *β*-catenin interact in the specialized cell-cell adhesion complexes between photoreceptor cells and Müller glia cells in the outer limiting membrane of the human retina (Nagel-Wolfrum et al., 2022).

In the cWnt signaling pathway, in the absence of the Wnt ligands, the levels of *β*-catenin in the cytoplasm are kept low by the destruction complex which is composed of the scaffold proteins axin and APC (adenoma polyposis coli), two kinases, GSK3b (glycogen synthase kinase 3b) and CK1 (casein kinase 1), and the protein phosphatase PP2A. Cytoplasmic *β*-catenin molecules are phosphorylated by the activity of the destruction complex and subsequently targeted for ubiquitination and proteosomal degradation (Kimelman and Xu, 2006) (see below summary Figures 8A, B).

Binding of Wnt ligands to Fz (Frizzled) receptors and the co-receptors LRP5/6 (low density lipoprotein receptor-related protein) at the plasma membrane triggers molecular events leading to inhibition of the destruction complex. Activated receptor complexes recruit Dvl (dishevelled) through direct binding which induces formation of the LRP-associated Wnt signalosomes (Bilic et al., 2007). Dvl, in turn, recruits conductin/axin and GSK3 kinase, destabilizing the *β*-catenin destruction complex and subsequently blocks the phosphorylation of *β*-catenin by GSK3 kinase activity (Zeng et al., 2008). Unphosphorylated *β*-catenin resists degradation, accumulates in the cytoplasm, and translocates into the nucleus. Nuclear *β*-catenin binds to transcription factors of the TCF/LEF (T cell factor/lymphoid enhancer factor) family. TCF/LEF proteins (hereafter referred to as TCF) bind to DNA but have a limited ability to activate transcription by their own. Binding of *β*-catenin leads to the formation of two-membered TCF/β-catenin transcriptional activators that convert Wnt signals into transcription of specific cWnt target genes (Archbold et al., 2012).

However, the cWnt signaling pathway and its regulation by *β*-catenin is much more complex, as briefly described above (Valenta et al., 2012). The function of *β*-catenin in cWnt is not only regulated by phosphorylation but can also be controlled by acetylation or by the binding of protein inhibitors. For example, the acetylation at lysine residue 49 (K49) of *β*-catenin can inhibit its degradation promoting the translocation of *β*-catenin into the nucleus and transcription of cWnt target genes (Ge et al., 2009; Liu et al., 2020). Binding of proteins like chibby (CBY1) and YWHAE (14-3-3ε) to the C-terminal domain of *β*-catenin blocks the *β*-catenin activation (Takemaru et al., 2009). Failure to suppress cWnt signaling often leads to developmental dysfunction and carcinogenesis, highlighting the important role of suppressor loops in cWnt signaling pathway (Kumar and Tanwar, 2017).

Here, we provide several lines of evidence that the USH1C protein harmonin is a suppressor of the cWnt signaling pathway by interacting with *β*-catenin. We demonstrate that overexpression of harmonin in HEK293T cells significantly reduces cWnt activation while a truncated form of harmonin does not. Furthermore, we found that cWnt signaling activity is significantly increased and that the expression of target genes and genes related to cWnt pathway are differentially expressed in harmonin-deficient *USH1C* patient-derived fibroblasts. Finally, the application of a translational read-through drug restores the cWnt signaling phenotype in *USH1C* ^R31*/R80Pfs*69^ (*USH1C*) patient-derived cells. Conclusively, we provide first molecular and functional evidence for a role of cWnt signaling in the development in human USH.

## Material and methods

### Antibodies

The following primary antibodies were used: mouse monoclonal anti-β-catenin (Santa Cruz, CA, United States; cs-7963; WB 1:500, IF 1:100), rabbit polyclonal anti-harmonin (H3) (custom-made Reiners et al., 2003); WB 1:1,000, IF 1:500), rabbit polyclonal anti-GFP (gift from Dr. W. Clay Smith; WB 1:500, IF 1:200), rabbit polyclonal Histon H3 (Cell Signaling Technology, Danvers, MA, United States; CST 4499T; WB 1:1,000), mouse monoclonal α-tubulin (Sigma-Aldrich, St Louis, MO, United States; T9026; WB 1:8,000, IF 1:800). We additionally used a rabbit monoclonal anti-*β*-catenin_K49 (lys49) (Cell Signaling Technology, Danvers, MA, United States; CST D7C2, Rabbit mAb #9030; WB 1:1,000, IF 1:200) commonly used for the analyses of *β*-catenin specifically acetylated at the residue 49 (e.g., Patnaik et al., 2019; Yamada et al., 2022). Secondary antibodies were conjugated to Alexa 488, Alexa 555, Alexa 568, or Alexa 647, respectively, purchased from Molecular Probes (Life Technologies, Darmstadt, Germany) or from Rockland Inc. (Gilbertsville, PA, United States). Nuclear DNA was stained with DAPI (4′,6-diamidino-2-phenylindole; 1 mg/mL) (Sigma-Aldrich, St Louis, MO, United States).

### DNA constructs

For transfection of HEK293T cells, a pcDNA-Dest47 vector (GFP at C-terminal) was used expressing the human harmonin a1 isoform or a mutant harmonin R31* cDNA. The nonsense mutation R31* was introduced into the wild-type construct by *in vitro* mutagenesis. The empty vector served as a control for all experiments except for the GFP-Trap. Here, an empty pcDNA-Dest53 (GFP at N-terminal) was used as a control. The pMS`BC-*β*-catenin plasmid was used for the expression of human *β*-catenin (Miyatani et al., 1992).

### Translational read-through inducing drugs (TRIDs)

Ataluren (PTC124; Absource Diagnostics GmbH, Munich, Germany) was dissolved in dimethyl sulfoxide (DMSO; Sigma-Aldrich, St Louis, MO, United States). For treatment, 5 μg/mL and 10 μg/mL of Ataluren were used. DMSO (0.2%) served as control.

### Cell culture of HEK293T cells

HEK293T cells were cultured in Dulbecco’s modified Eagle’s medium (DMEM) with Glutamax containing 10% heat-inactivated fetal bovine serum (FBS) (Thermo Fisher Scientific, Waltham, MA, United States) at 37°C in 5% CO_2_. Transfection of cells was performed using GeneJuice^®^ (Merck Millipore, Darmstadt, Germany) according to manufacturer’s instructions. Ataluren was applied 6 h after transfection. Cells were used for luciferase assays 48 h after Ataluren treatment.

### Human primary fibroblast and cultures

Dermal primary fibroblast lines were expanded from skin biopsies of human subjects (ethics votume: Landesärztekammer Rhineland-Palatinate to KNW). We analysed dermal fibroblasts of a male patient genetically diagnosed for USH1 with biallelic *USH1C* mutations c.91C>T; p.(R31*) and c.238dupC; p.(R80Pfs*69) (*USH1C* ^R31*/R80Pfs*69^) (Nagel-Wolfrum et al., 2022), as well as control fibroblasts from a healthy proband. Cells were cultured in DMEM containing 10% heat-inactivated FBS (Thermo Fisher Scientific, Waltham, MA, United States) at 37°C in 5% CO_2_. Ataluren was applied for 24 h in the appropriate medium.

### Stimulation of Wnt signaling pathway

For activation of the Wnt signaling pathway, HEK293T and fibroblasts were either stimulated by overexpression of *β*-catenin [*β*-cat-stimulation (*β*-catS)] (Lin et al., 2006; Liu et al., 2020) transfecting *β*-catenin (pMS`BC_*β*-catenin human) or alternatively, by culturing cells for 24 h in Wnt conditioned media [Wnt medium stimulation (WMS)] (Cruciat et al., 2010; Patnaik et al., 2019). Wnt conditioned medium was collected from L Wnt-3A cells according to manufacturer’s protocol (CRL-2647 ATCC, Manassas, VA, United States). It should be noted that in *β*-catS the overexpression of *β*-catenin increases cytoplasmic *β*-catenin, which saturates the *β*-catenin destruction complex, and that the large pool of free *β*-catenin activates the cWnt pathway far more effectively than Wnt ligands in Wnt conditional medium. This is due to the fact that *β*-catS activation is independent of the concentration of Wnt ligands, the number of available endogenous *β*-catenin molecules in the cell, and the number of Wnt surface receptor complexes at the plasma membrane.

### Luciferase assay

Analysis of the Wnt/*β*-catenin signaling pathway activity was performed using the Dual-Glo^®^ Luciferase Assay System (Promega, Madison, WI, United States) according to the manufacturer’s protocol. Briefly, HEK293T cells were seeded into a 96-well plate and after 24 h co-transfected with TopFlash (Firefly luciferase) for determining the activity of TCF/LEF dependent Firefly luciferase expression, and pRL-TK (Renilla luciferase) for normalization of the transfection efficacy, pcDNA-Dest47 vectors, namely pcDNA-Dest47-Harm_a1_GFP, pcDNA-Dest47-Harm_R31*_GFP, pcDNA-Dest47_GFP and additionally *β*-catenin (pMS`BC_*β*-catenin human) for stimulation. Alternatively, cells were stimulated with Wnt conditioned media 24 h post-transfections (Cruciat et al., 2010; Patnaik et al., 2019). Luciferase activity was measured after 24 h in a Varioskan Flash plate reader (Thermo Fisher Scientific, Waltham, MA, United States). Firefly luciferase activity was normalized to Renilla luciferase activity in each well. Background subtraction was done using values of medium only.

### Immunocytochemistry

HEK293T cells as well as human fibroblasts were washed with PBS prior to fixation with 2% paraformaldehyde (PFA) for 15 min (Samanta et al., 2019). Fixed cells were washed twice with phosphate-buffered saline (PBS) and permeabilized with 0.1% TritonX-100 for 10 min. After short washing with PBS, specimens were incubated for at least 45 min in blocking solution (0.5% cold-water fish gelatin, 0.1% ovalbumin in PBS). Incubation with primary antibodies was done overnight at 4°C. The next day, after washing with PBS, samples were incubated with secondary antibodies and DAPI for 2 h in the dark at room temperature. After washing, coverslips were mounted in Mowiol (Roth, Karlsruhe, Germany).

### Western blot analysis

For lysis, cells were incubated with Triton X-100 lysis buffer (50 mM Tris–HCl pH 7.5, 150 mM NaCl, and 0.5% Triton X-100) containing protease inhibitor cocktail (PI mix; Roche, Basel, Switzerland) and sonified. Cell lysates samples were mixed with SDS-PAGE loading buffer (10% glycerin, 250 mM Tris-base, 2% SDS, 0.5 mM EDTA, 0.001% bromophenol blue, HCL pH 8.5) and separated on self-made 12% or 15% polyacrylamide gels, first for 5 min at 110 V, then at least for 60 min at 180 V using the Mini-PROTEAN System (Bio-Rad Laboratories, Feldkirchen, Germany). For Western blot (WB) analyses, proteins were transferred to polyvinylidene difluoride membranes (Millipore, Schwalbach, Germany) in blotting buffer (25 mM TRIS, 192 mM glycine, 0.025% (v/v) SDS, 20% (v/v) methanol) for 75 min at 100 V using the Mini Trans-Blot Electrophoretic Transfer Cell (Bio-Rad Laboratories, Feldkirchen, Germany). After blocking the membrane with Applichem blocking reagent (Applichem, Darmstadt, Germany) for 1–2 h at room temperature, immunoreactivities were detected by applying primary and appropriate secondary antibodies, Alexa Flour 680 (Invitrogen) or IR Dye 800 (Rockland, Gilbertsville, United States), employing the Odyssey infrared imaging system (LI-COR Biosciences, Lincoln, NE, United States) and processed *via* Fiji.

### GFP-Trap^®^

Magnetic GFP-Trap^®^ agarose beads (Chromotek, Planegg, Germany; gtma-20) were used for immunoprecipitation assays according to the manufacture’s protocol. In brief, GFP-tagged *USH1C*/harmonin proteins as well as *β*-catenin were expressed in HEK293T cells for 48 h. For lysis, cells were incubated with Triton X-100 lysis buffer (50 mM Tris–HCl pH 7.5, 150 mM NaCl, and 0.5% Triton X-100) containing protease inhibitor cocktail (PI mix; Roche, Basel, Switzerland) and sonified. Cells were lysed as described above and 10% of total cell lysate was separated for input fraction. Beads were equilibrated with 10 mM Tris-HCl pH 7.5, 150 mM NaCl, 0.5 mM EDTA dilution buffer and incubated with remaining lysates of *β*-catenin and GFP-tagged harmonin or GFP only for 2 h at 4°C under constant rotation. After washing of beads with dilution buffer, precipitated proteins were eluted with Laemmli buffer and analyzed on SDS-PAGE (12% polyacrylamide gels) followed by Western blot (WB).

### Proximity ligation assay (PLA)

PLAs were performed in HEK293T cells using the *in-situ* proximity ligation assay Duolink PLA probes anti-rabbitPLUS, anti-mouseMINUS, and Detection Reagent Red (Sigma-Aldrich, St Louis, MO, United States) as previously described in (Sorusch et al., 2017; Yildirim et al., 2021). Briefly, cells were fixed and incubated with primary antibodies overnight at 4°C. After washing, samples were incubated with oligonucleotide-labelled secondary antibodies (“PLA probes”) for 1 h at 37°C. Several washing steps were followed by ligation for 30 min at 37°C using hybridizing connector oligonucleotides. After washing, samples were incubated with amplification reagents for 100 min, followed by addition of fluorescent-labelled oligonucleotides. For the negative controls only one protein specific antibody was used or none of them. Coverslips were mounted in Mowiol (Roth, Karlsruhe, Germany).

### Microscopy and image processing

Immunofluorescence staining was documented and analyzed on a Leica DM6000B microscope using LAS-AF software (Leica, Bensheim, Germany). Objectives used were ×40 and ×63. Western blots were analyzed using the Odyssey infra-red imaging system (LI-COR Biosciences, Lincoln, NE, United States). Image processing was done with ImageJ/Fiji software (Schindelin et al., 2015).

### Statistical analysis and quantifications

Analysis of *β*-catenin_K49 intensity in immunofluorescence staining in fibroblasts was performed using a script in ImageJ/Fiji software (Schindelin et al., 2015). Briefly, the nucleus area was identified by DAPI staining followed by a measurement of *β*-catenin_K49 intensity in this area (µm^2^). PLA signals were identified with an ImageJ Fiji script by measuring the number of particles in the region of interest (ROI) processed picture. Pearson correlation coefficient was defined *via* Fiji using the colocalization Coloc 2 plugin (https://imagej.net/plugins/coloc-2). For Western blot analysis, relative band intensities were normalized to the relative band intensities of the corresponding control. Quantification of results was performed in MS-Excel using Student’s *t*-test (unpaired, two-tailed, assuming equal variance). Error bars are represented as standard deviation. At least three independent experiments were performed. The significance levels were set when *p* < 0.05 (*), *p* < 0.01 (**), *p* < 0.001 (***).

### Fiji/ImageJ script: Fluorescence intensity analysis

open(“...”);
run(“Set Scale...”, “distance = 0.495 known = 1 unit = µm global”);
selectWindow(“Image001DMEM_ctr_ch03.tif”); run(“8-bit”);
setAutoThreshold(“Default dark no-reset”);
setOption(“BlackBackground”, false);
run(“Convert to Mask”); run(“Watershed”);
run(“Analyze Particles...”, “size = 2000-Infinity pixel circularity = 0.50–1.00 show = Overlay display exclude clear summarize add”);
close(); selectWindow(“Image001DMEM_ctr_ch01.tif”);
roiManager(“Show None”);
roiManager(“Show All”);
roiManager(“Measure”);
run(“Flatten”);

### Fiji/ImageJ script: Analysis of fluorescent PLA signals

open(“...”);
run(“8-bit”);
run(“Bandpass Filter...”, “filter_large = 40 filter_small = 3 suppress = None tolerance = 5 autoscale saturate”);
//run(“Brightness/Contrast...”);
run(“Apply LUT”);
setAutoThreshold(“Otsu dark no-reset”);
//run(“Threshold...”);
//setThreshold(89, 255);
setOption(“BlackBackground”, false);
run(“Convert to Mask”);
run(“Analyze Particles...”, “ circularity = 0.20–1.00 show = Overlay display exclude clear summarize add”);
run(“Flatten”);

### Fractionation of cell compartments

The isolation of cytosol and nucleus fractions was performed as described for the first steps by (Yildirim et al., 2021). Briefly, HEK293T cells were transfected using GeneJuice^®^ (Merck Millipore, Darmstadt, Germany). After 24 h medium was changed to fresh DMEM or Wnt medium. The next day, cells were harvested and centrifuged in PBS. Cell pellets were resuspended in 1.25 packed cell volume of hypotonic MC buffer (10 mM HEPES pH 7.6, 5 mM MgAc_2_, 10 mM KAc, 1 mM DTT, 1x protease inhibitor) and left on ice for 15 min. For lysis, a 1-mL syringe with G25 needle was used. For separation of nucleus and cytosol, lysed cells were centrifuged at 1,500 g for 5 min. The supernatant was taken (cytoplasmic fraction) and the nucleus pellet was incubated in Triton X-100 lysis buffer (50 mM Tris–HCl pH 7.5, 150 mM NaCl, and 0.5% Triton X-100) containing protease inhibitor cocktail (PI mix; Roche, Basel, Switzerland) and sonified. Protein lysates were separated by SDS-PAGE gel electrophoresis (15% polyacrylamide gels), followed by Western blotting as described above. Before blocking, blots were incubated and stained with Revert 700 total protein Stain pack (LI-COR Biosciences, Lincoln, NE, United States) for visualizing of total protein amount.

### RNA-Sequencing

For RNAseq analysis human *USH1C* ^R31*/R80Pfs*69^ (*USH1C*) patient-derived and healthy control fibroblast cell lines were used. Human fibroblasts were maintained in either DMEM 10% FBS (Thermo Fisher Scientific, Waltham, MA, United States) or Wnt-3A conditioned medium (ATCC, Manassas, VA, United States) for 24 h at 37°C, 5% CO_2_ before RNA isolation. RNA isolation was performed using the RNeasy Mini Kit by following the manufacturer’s instructions (Qiagen, Hilden, Germany). Subsequent RNA library preparation and whole transcriptome RNAseq were conducted by Novogene using the Illumina NovaSeq 6000 Sequencing System (Novogene, Cambridge, UK). Following quality control, genes with an adjusted *p*-value <0.05 were considered as significantly differentially expressed (Novogene, Cambridge, UK). Differentially expressed Wnt signaling pathway and Wnt target genes were identified by manual literature research (Herbst et al., 2014; Ramakrishnan and Cadigan, 2017; Ghahramani et al., 2018; Lecarpentier et al., 2019; Boonekamp et al., 2021) and expanded by Gene Ontology annotations from the EBI’s GOA database (https://www.ebi.ac.uk/QuickGO/, query 07.09.2022 with the gene ontology (GO)-terms Wnt pathway (GO:0016055) and canonical Wnt pathway (GO:0060070) (Binns et al., 2009). For those genes, the transformed FPKM-values were clustered in a heatmap for each cellular treatment. Heatmaps were generated by using Heatmapper (http://www.heatmapper.ca/08.12.2022) (Ashburner et al., 2000; Mi et al., 2019; Gene Ontology Consortium, 2021). Additional GO Enrichment Analysis was conducted for the previously identified Wnt target genes using ClueGO v2.3.3.

### *In silico* missense variant analysis of *USH1C* nonsense mutation

*In silico* prediction tools were used to assess whether amino acid substitutions after translational read-through of the R31*-mutant *USH1C*/harmonin protein are expected to have an impact on the protein. All variants were denoted based on the NCBI reference sequence for *USH1C* (NM_153676.4; GRCh38 and ENSG00000006611.11). Variant classification was assessed using the following computational *in silico* tools: PolyPhen-2 and tools integrated into Alamut Visual Plus v.1.1 (Sophia Genetics) using default settings. PolyPhen-2 (Polymorphism Phenotyping v2) was used, which predicts the possible impact of an amino acid substitution on the structure and function of a human protein using straightforward physical and comparative considerations (Adzhubei et al., 2010), and scores range from 0 (benign) to 1 (probably damaging). PhyloP determines the evolutionary conservation and acceleration of a given nucleotide, and positive scores are assigned to sites predicted to be conserved whereas negative scores indicate a fast-evolving site (Pollard et al., 2010). The Grantham distance scores missense substitutions regarding the physicochemical difference between the exchanged amino acids with scores ranging from 0 to 215 (Grantham, 1974). Align-GVGD (v2007) provides prediction classes defining a spectrum of classifications with C0 suggesting least likely to interfere with protein function to C65 being most likely to affect functionality of the protein (https://agvgd.iarc.fr/index.php) (Tavtigian et al., 2006). SIFT (Sorting Intolerant From Tolerant, v4.0.3/v6.2.0) applies a score range of 0.0 for a deleterious effect to 1.0 for the substitution being tolerated (http://sift.bii.a-star.edu.sg/) (Kumar et al., 2009). MutationTaster (v2013) classifies the variants either as “disease-causing” or as a “polymorphism” with the *p*-value indicating the certainty of the prediction (http://www.mutationtaster.org/) (Schwarz et al., 2010).

## Results

### Harmonin (USH1C) interacts with *β*-catenin *in vitro* and *in situ* in cells

The binding of harmonin and *β*-catenin has been identified in a yeast two-hybrid screen by (Johnston et al., 2004) but could not be validated by *in vitro* interaction assays at that time. More recently, we have shown in *in vitro* GST pull-down experiments applying bacterially expressed proteins that *β*-catenin can bind *via* its PDZ binding motif (PBM) in its very C-terminal tail to the PDZ1 and PDZ3 domains of harmonin (Nagel-Wolfrum et al., 2022). Here, we tested the interaction of harmonin with *β*-catenin by complementary GFP-Trap immunoprecipitation assays from lysates of mammalian HEK293T cells expressing GFP-tagged harmonin_a1 and untagged *β*-catenin (Figure 1B). Western blots showed that *β*-catenin was recovered in the GFP-Traps with harmonin-GFP, but not with GFP alone.

To proof whether these interactions can also occur between the endogenous proteins *in situ* in cells, we performed proximity ligation assays (PLAs) in HEK293T cells. Fluorescence microscopy revealed positive harmonin-*β*-catenin PLA signals in the cytoplasm and the nucleus indicating close proximity of both proteins (Figure 1C). Quantification of PLA signals demonstrated that the harmonin-*β*-catenin PLA signals were 3-4-fold higher when compared to all control PLAs (Figure 1D; Supplementary Figure S1).

### Harmonin also interacts with the acetylated form of *β*-catenin

**FIGURE 2:**
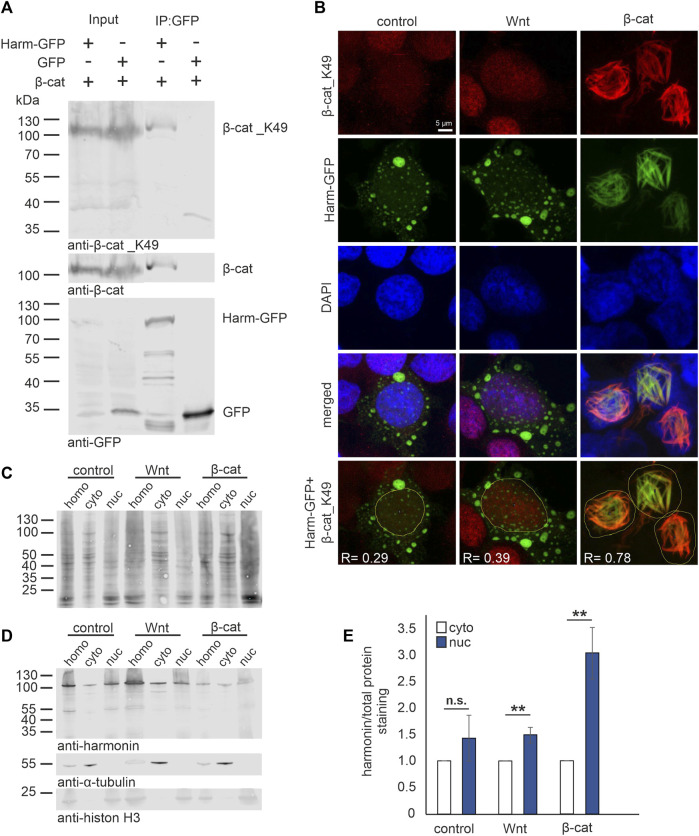
Harmonin interaction with acetylated *β*-catenin_K49 in the nucleus. **(A)** Western blot analysis of a representative GFP-Trap^®^ pull down shows the interaction between harmonin a1-GFP (Harm-GFP) and acetylated *β*-catenin_K49 (*β*-cat_K49). HEK293T cells were transfected with *β*-cat and Harm-GFP or GFP alone, respectively. Harm-GFP but not GFP alone was able to precipitate *β*-catenin (*β*-cat) as well as *β*-cat_K49; 12% polyacrylamide gel; N = 3 experiments. **(B)** Immunofluorescence localization of Harm-GFP (green) and *β*-cat_K49 (red) under different stimulation. HEK293T cells were transfected with Harm-GFP and stimulated for Wnt signaling with Wnt medium (Wnt) or transfection of *β*-cat. DAPI (blue) marks the nucleus. In unstimulated (control) state (left panel) (Pearson correlation coefficient: Harm-GFP and *β*-cat_K49: R = 0.29; *n* = 136 cells; N = 3 experiments) and after Wnt stimulation (middle panel) (Pearson correlation coefficient: Harm-GFP and *β*-cat_K49: R = 0.39; *n* = 134 cells; N = 3 experiments), Harm-GFP is distributed in nucleus and cytosol of the cell. The *β*-cat_K49 staining is almost absent in the unstimulated state, however, there is an increase in anti-*β*-cat_K49 fluorescence in the nucleus after Wnt stimulation. Additional transfection with *β*-cat (left panel) results in *β*-cat_K49 bundles in the nucleus with which Harm-GFP colocalizes. Analysis of Pearson correlation coefficient (lowest panel) reveals strong interaction between Harm-GFP and *β*-cat_K49 with value of R = 0.78 (*n* = 169 cells; N = 3 experiments). **(C–E)** Western blot analysis of cell fractions (30 μg total protein loaded), separated from homogenate (homo) in cytosol (cyto) and nucleus (nuc). HEK293T cells were transfected with Harm-GFP and stimulated for Wnt signaling with Wnt medium or transfection of *β*-cat. Unstimulated HEK293 cells served as control. **(C)** Staining of total protein amount was compared to **(D)** Harm-GFP staining in the fractions; *α*-Tubulin marks the cytosol fraction and histon H3 the nucleus fraction (15% polyacrylamide gel). Statistical analysis **(E)** reveals a non-significant increase of 1.4-fold of harmonin in unstimulated cells (control) but a significant increase of 1.5-fold comparing nuclear to cytosolic harmonin (set to 1) in Wnt stimulated cells. Co-transfection with *β*-cat results in a significant increase of 3-fold of nuclear harmonin. Two-tailed Student’s *t*-test, **p ≤ 0.01; N = 3 experiments.

*β*-catenin is a transcriptional coactivator of TCF/LEF target genes in the canonical Wnt (cWnt) signaling pathway and its transcriptional activity is highly regulated by the formation of the *β*-catenin destruction complex (Valenta et al., 2012). The acetylation at the lysin 49 residue stabilizes *β*-catenin (*β*-catenin_K49) and inhibits the formation of the *β*-catenin destruction complex and its degradation. Moreover, the acetylation promotes the nuclear translocation of *β*-catenin_K49 and thereby the activation of the cWnt signaling pathway (Liu et al., 2020). To test whether harmonin also binds to the acetylated *β*-catenin we probed Western blots of harmonin GFP-Traps with pan *β*-catenin and *β*-catenin_K49 specific antibodies (Figure 2A). Both, *β*-catenin and *β*-catenin_K49 were recovered in GFP-Traps with harmonin-GFP, but not with GFP alone indicating that harmonin also binds the acetylated *β*-catenin.

### Acetylated *β*-catenin recruits harmonin into the nucleus after cWnt pathway stimulation

Next, we investigated whether the stimulation of cWnt pathway affects the subcellular distribution of *β*-catenin_K49 and harmonin. For this, we stimulated harmonin a1-GFP transfected HEK293T cells applying Wnt conditional medium [Wnt medium stimulation (WMS)] (Patnaik et al., 2019) or by overexpression of *β*-catenin [*β*-cat-stimulation (β-catS)] (Lin et al., 2006; Liu et al., 2020). We subsequently analyzed the localization of harmonin and *β*-catenin_K49 in the cells by immunocytochemistry *in situ* and biochemically by Western blots of cell fractions (Figures 2B–E). Confocal analysis of *β*-catenin_K49 in unstimulated state (control) was almost absent due its degradation by the activity of the *β*-catenin destruction complex (Kimelman and Xu, 2006). However, after stimulation of Wnt pathway, especially after *β*-catenin transfections, acetylated *β*-catenin immunofluorescence was found in the nucleus (Figure 2B, upper panel) (see, Liu et al., 2020). Anti-GFP immunofluorescence staining showed that harmonin a1-GFP was mostly localized in the cytoplasm in cells cultured in normal culturing medium (Figure 2B, control, left panel). After the application of WMS in addition to the cytoplasmic localization some harmonin was also found in the nucleus (Figure 2B, Wnt, middle panel). In contrast, after *β*-catenin co-transfection nearly all harmonin translocated into the nucleus. Nuclear harmonin co-localized with *β*-catenin_K49 at filamentous structures (Figure 2B, *β*-cat, right panel). Such *β*-catenin filament bundles have been described previously in nuclei of MDKC cells overexpressing *β*-catenin (Simcha et al., 1998). Analysis of Pearson correlation coefficient in cells co-transfected with harmonin and *β*-catenin revealed an average positive value of 0.78 and therefore, a strong linear relation between *β*-catenin_K49 and harmonin indicating the interaction of both proteins at the nuclear filament bundles.

Translocation of harmonin into the nucleus caused by Wnt stimulation was also confirmed in cellular fractions. To this end, Western blots were first stained for total protein (Figure 2C) and subsequently for harmonin by the H3 antibody against harmonin (Figure 2D). We found harmonin under all conditions (unstimulated control, WMS and *β*-catS) in both the cytosol and the nucleus fractions. However, when we calculated the ratio of harmonin in cellular fractions and total protein, we observed significantly more harmonin in the nuclear fraction after Wnt stimulation, *via* WMS as well as *β*-catS (Figure 2E). Especially in *β*-catS stimulated cells the amount of harmonin was increased more than 3-fold in the nuclear fraction when compared to the cytosolic fraction.

In summary, these results showed that harmonin translocates together with *β*-catenin/*β*-catenin_K49 into the nucleus when the availability of *β*-catenin and/or the activity of cWnt signaling is increased.

### Harmonin expression decreases Wnt signaling activity

**FIGURE 3:**
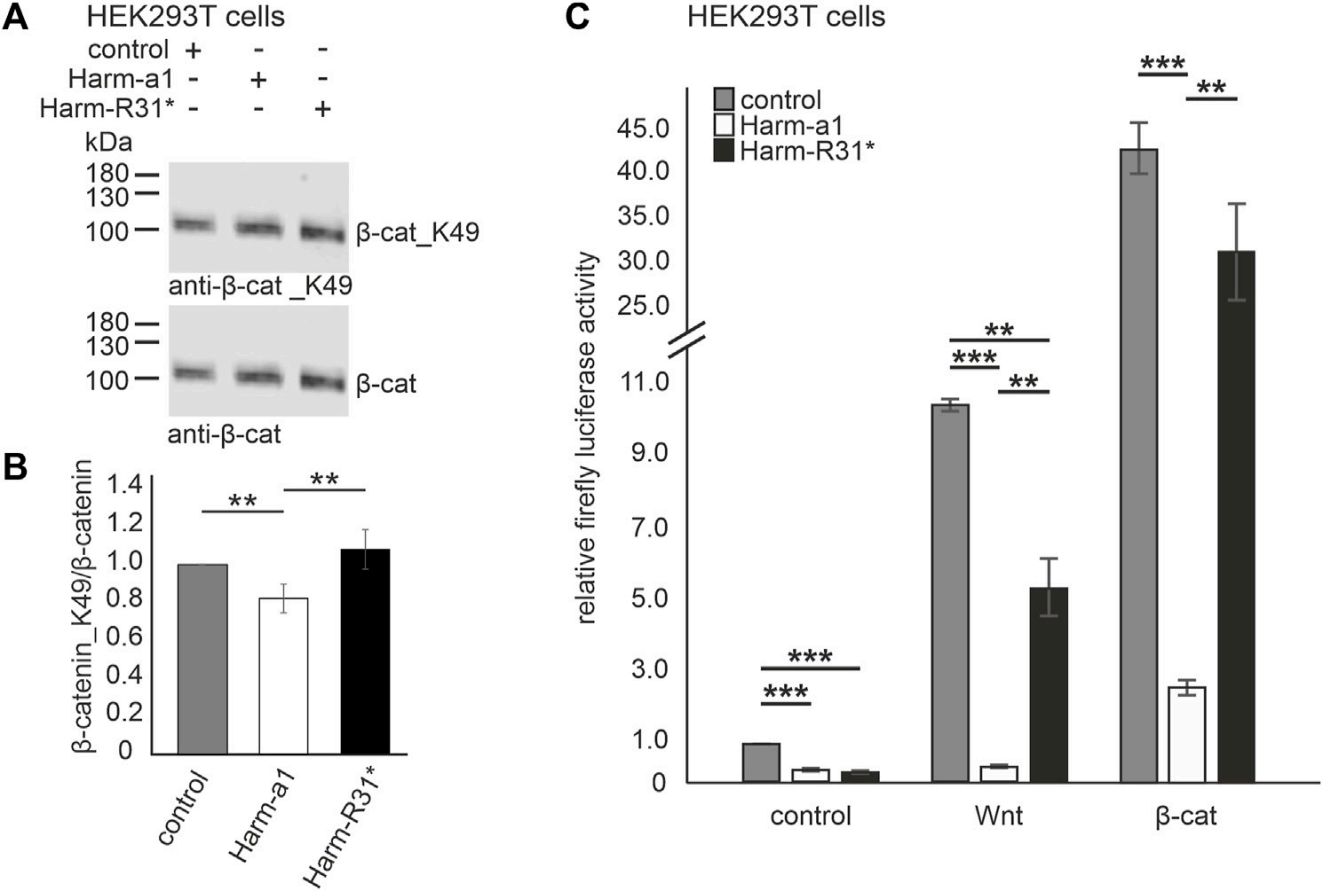
Harmonin overexpression leads to the decrease in Wnt signaling activity **(A)** Representative Western blot analysis of *β*-catenin (*β*-cat) expression in HEK293T cells transfected with harmonin a1-GFP (Harm-a1), truncated harmonin a1_R31*-GFP (Harm-R31*), or GFP (control) and *β*-catenin (*β*-cat). **(B)** Quantification reveals that the expression of full length harmonin a1 significantly decreases *β*-cat_K49/*β*-cat ratio (−0.2) while the expression of truncated harmonin a1-R31* did not alter the *β*-cat_K49/*β*-cat ratio when compared to control transfected HEK293T cells; 12% polyacrylamide gel. Two-tailed Student’s *t*-test, **p ≤ 0.01; N = 5 experiments. **(C)** Wnt response in transfected HEK293T cells measured by TCF/LEF luciferase activity assay. In unstimulated condition (control, left) luciferase activity is significantly decreased in Harm-a1 and Harm-R31* transfected cells compared to empty vector control. Stimulation with Wnt conditioned medium leads to an increase in luciferase activity for all, whereby control as well as Harm-R31* show significantly more cWnt signaling activity than Harm-a1 (Wnt, middle). Addition of *β*-cat results in the highest Wnt activity and reveals the largest difference of luciferase activity between Harm-a1 to control and Harm-R31* (*β*-cat, right). cWnt signaling is significantly decreased in Harm-a1 transfected cells. Two-tailed Student’s *t*-test, **p ≤ 0.01, **p ≤ 0.001; N = 3 experiments (three triplicates each condition).

Next, we tested whether *USH1C*/harmonin expression alters the activation cWnt signaling pathway. For this, we overexpressed GFP-tagged human harmonin a1 (Harm-a1) in HEK293T cells in which the cWnt signaling was stimulated by overexpression of *β*-catenin (*β*-catS). The quantification of Western blots revealed that the ratio of *β*-catenin_K49/*β*-catenin was significantly reduced in harmonin overexpressing cells compared to control transfected cells (Figures 3A, B). This weak, ∼0.23-fold reduction was not observed in parallel experiments overexpressing the truncated harmonin a1_R31* version (Harm-R31*) with a disease-causing nonsense mutation that results in the expression of a severely truncated harmonin a1 protein (Goldmann et al., 2010; Goldmann et al., 2011; Goldmann et al., 2012). These results indicated a negative regulation of cWnt signaling by harmonin.

To verify these promising results, we next analyzed cWnt activity using a cWnt signaling luciferase assay sensing of TCF/LEF transcription factor activity (see Material and Methods). For this HEK293T cells were transfected with Dual-Glo^®^ Luciferase vectors and GFP-tagged human harmonin a1 (Harm-a1), its harmonin a1_R31* (Harm-R31*) version or GFP alone, respectively and the relative firefly luciferase activity was determined (Figure 3C). Without Wnt stimulation the expression of both Harm-a1 and Harm-R31* led to a significant decrease in the luciferase activity compared to the mock transfections (Figure 3C, left panel). In mock transfected cells, the stimulation of the cWnt pathway by both Wnt conditional medium (Wnt medium stimulation (WMS)) or by overexpression of *β*-catenin (*β*-catS) led to drastic increases of the relative firefly luciferase activity, ∼10-fold and over 40-fold, respectively, indicating the high sensitivity of the assay and strong cWnt pathway activations under both stimulation conditions. Harm-a1 overexpression completely extinguished the relative firefly luciferase activity under conditions of stimulation by Wnt medium and significantly reduced the increase from 40-fold to less than 3-fold when cWnt signaling was stimulated by *β*-catenin. In contrast, the truncated Harm-R31* version reduced the relative firefly luciferase activity nearly to half under conditions of stimulation by Wnt medium compared to the GFP control and no significant difference in the relative luciferase activity between Harm-R31* and GFP controls was observed when cWnt signaling was stimulated by *β*-catenin. Taken together, our results obtained with the cWnt luciferase assay confirm that *USH1C*/harmonin suppresses cWnt signaling in HEK293T cells.

### The expression of cWnt target genes and cWnt pathway genes in USH1C patient-derived fibroblasts

Next, we aimed to validate our results obtained in HEK293T cells in USH1C patient-derived dermal fibroblasts cells, which have recently been established as a cellular model of USH disease for translational research (Grotz et al., 2022; Nagel-Wolfrum et al., 2022). In human dermal fibroblasts, Wnt signaling is important for the cellular function related to hair follicle development, regional cell identity, and wound healing, and its dysregulation can lead to skin fibrosis or wound healing defects (Enzo et al., 2015; Griffin et al., 2022). We cultivated dermal fibroblasts from a clinically characterized USH1C patient with *USH1C* ^R31*/80Pfs*69^ (hereafter referred to as USH1C fibroblasts) and a healthy control individual (healthy fibroblasts) in control medium and Wnt conditioned medium (WMS), respectively, and examined their transcriptomes by RNAseq.

**FIGURE 4:**
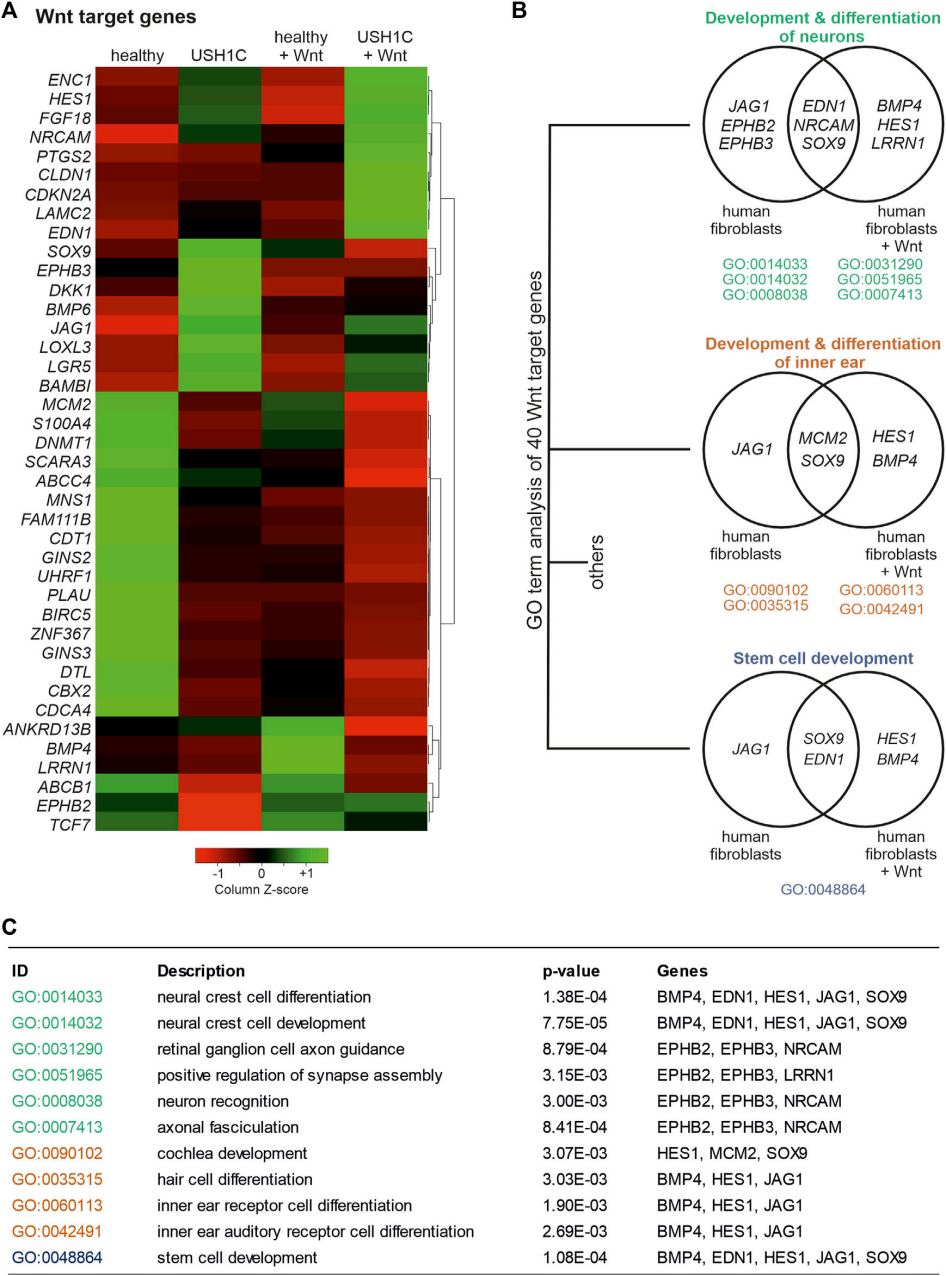
RNAseq analysis uncovers differential expression of Wnt target genes in *USH1C* ^R31*/R80Pfs*69^ patient-derived fibroblasts. **(A)** Heatmap shows transformed FPKM-values scaled by z-score identifying 40 differentially regulated Wnt target genes in unstimulated and Wnt stimulated healthy (column 1 and 3) and *USH1C* ^R31*/R80Pfs*69^ patient-derived (*USH1C*) fibroblasts (column 2 and 4). Red areas correspond to a relative downregulation in gene expression, whereas green areas represent a relative upregulation. **(B)** GO enrichment analysis showing dysregulated Wnt target genes to be critical for distinct biological processes, such as “development and differentiation of neurons,” “development and differentiation of the inner ear,” as well as “stem cell development.” **(C)** Detailed description of GO terms summarized in **(B)**.

Principal Component Analysis (PCA) revealed a large distance in clustering of healthy and USH1C fibroblasts (Pearson correlation coefficient 0.865) indicating a great transcriptomic difference (Supplementary Figure S2A). Interestingly, WMS had a greater effect on the USH1C fibroblast transcriptome (Pearson correlation coefficient 0.796), as they clustered farther apart from each other when compared to their healthy counterparts (Pearson correlation coefficient 0.869). The greater transcriptomic difference of WMS on USH1C fibroblasts was further observed by differential expressed gene (DEG) analysis. For USH1C fibroblasts, WMS resulted in 5,303 DEGs when compared to their untreated counterparts (Supplementary Figure S2B; Supplementary Tables S1, S2), thereby including 23 genes that have been previously identified as Wnt targets (Herbst et al., 2014; Ramakrishnan and Cadigan, 2017; Ghahramani et al., 2018; Lecarpentier et al., 2019; Boonekamp et al., 2021) (Figure 4A, column 2 and 4). For healthy fibroblasts, WMS revealed only 2,590 DEGs (Supplementary Figure S2B; Supplementary Tables S1, S3) when compared to the untreated ones, comprising 16 Wnt target genes (Figure 4A, column 1 and 3). To further assess the consequence of the *USH1C* ^R31*/80Pfs*69^ mutations on transcriptome level, we additionally examined DEGs of healthy and USH1C fibroblasts. Consistent with the previous findings, WMS led to an increased amount of DEGs. Specifically, we were able to identify 3,265 DEGs (1,583 upregulated and 1,682 downregulated in USH1C + Wnt), including 31 Wnt target genes when comparing healthy and USH1C Wnt stimulated fibroblasts [Figure 4A, column 3 and 4, (Supplementary Figure S2B; Supplementary Tables S1, S4)], whereas untreated fibroblasts showed 2,229 DEGs (1,171 upregulated and 1,058 downregulated in USH1C), including 32 Wnt target genes [Figure 4A column 1 and 3, (Supplementary Figure S2B; Supplementary Tables S1, S5)]. Next, we performed Gene Ontology (GO) enrichment analysis to assess biological processes in which the identified dysregulated Wnt target genes are participating in. In total, differential expressed Wnt target genes were enriched for 126 biological processes (Supplementary Table S6). Of those, 11 GO terms revealed changes in “development and differentiation of neurons” (Figures 4B, C, green), “development and differentiation of the inner ear” (Figures 4B, C, orange), as well as “stem cell development” (Figures 4B, C, grey) when comparing healthy and USH1C fibroblasts and their WMS counterparts. Interestingly, SOX9, which is also known to be a regulator of retinogenesis and homeostasis in the adult retina, accounts for all three developmental GO terms regardless of whether fibroblasts were Wnt stimulated or not (Poche et al., 2008; Wu et al., 2022). DEG analysis also revealed SOX9 to be dysregulated when comparing untreated and Wnt treated USH1C fibroblasts, healthy and USH1C fibroblasts, as well as Wnt treated healthy and USH1C, but not for untreated and Wnt treated healthy fibroblasts.

**FIGURE 5:**
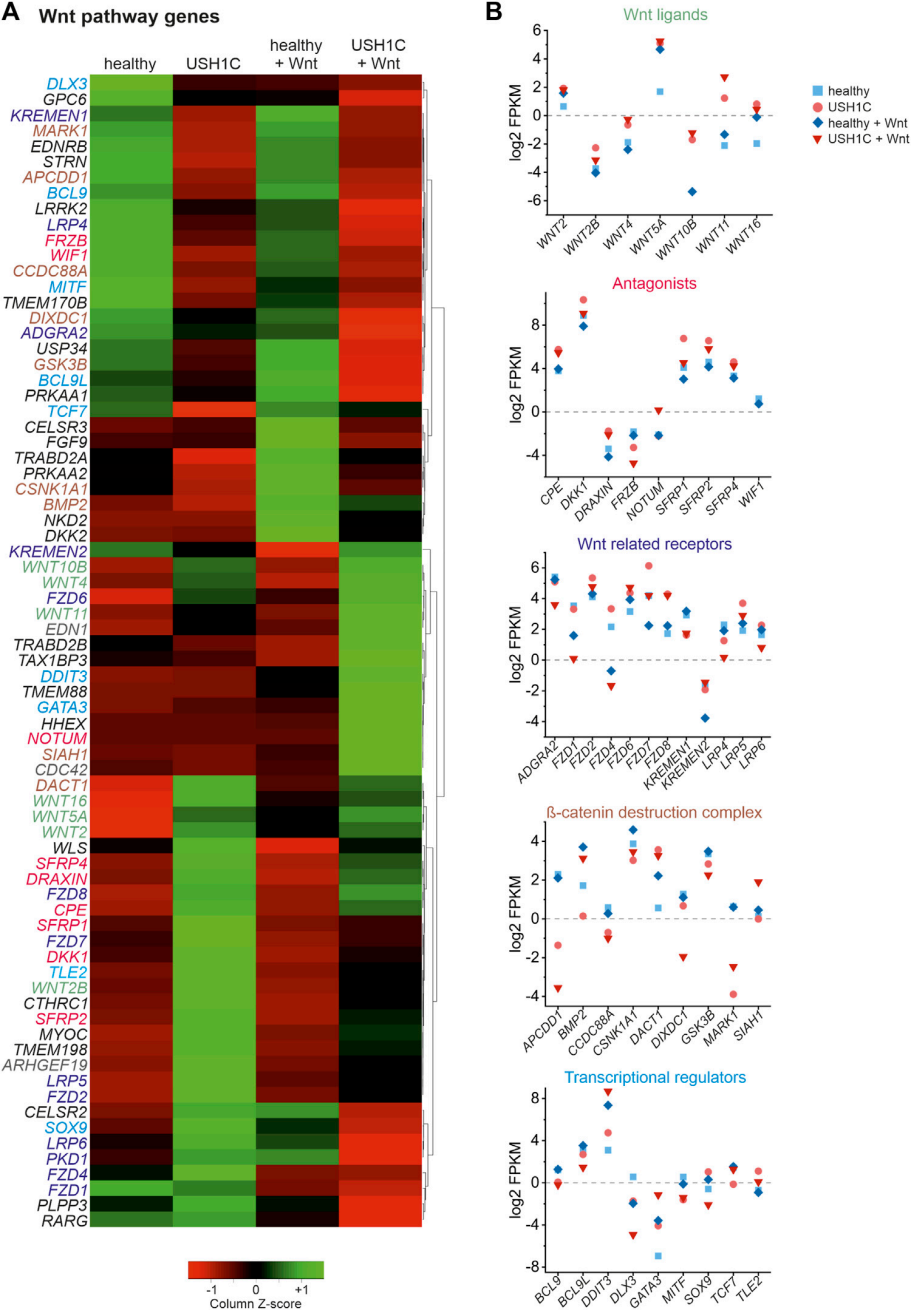
RNAseq reveals differential expression of Wnt signaling pathway genes in *USH1C* ^R31*/R80Pfs*69^ patient-derived fibroblasts. **(A)** Heatmap showing transformed FPKM-values scaled by z-score identifying 74 differentially regulated Wnt signaling pathway genes in unstimulated and Wnt stimulated healthy (column 1 and 3) and *USH1C* ^R31*/R80Pfs*69^ patient-derived (*USH1C*) fibroblasts (column 2 and 4). Red areas correspond to a relative downregulation in gene expression, whereas green areas represent a relative upregulation. **(B)** Log2 transformed FPKM-values show the expression levels of Wnt pathway genes blotted due to their area of action within the Wnt signaling pathway for unstimulated and Wnt stimulated healthy and USH1C^R31*/R80Pfs*69^ (USH) fibroblasts, respectively.

As we were able to observe substantial dysregulation, particularly of the expression of Wnt target genes, we next sought to elucidate the Wnt signaling pathway itself. To do so, differential gene expression of Wnt pathway genes was examined in unstimulated and Wnt stimulated healthy and USH1C fibroblasts, respectively. Wnt pathway genes were defined as those associated to the GO terms Wnt pathway (GO:0016055) and canonical Wnt pathway (GO:0060070). In total, we were able to identify and cluster 74 DEGs (Figure 5A). Categorization according to the site of action of gene products within the Wnt signaling pathway revealed five distinct categories (Figure 5B), namely Wnt ligands (Figure 5B, green, seven genes), secreted antagonists (pink, nine genes), Wnt related membrane receptors (Figure 5B, purple, 13 genes), *β*-catenin destruction complex (brown, nine genes) and transcriptional regulators (Figure 5B, blue, nine genes). Additional 27 genes are marked in black as these could not be clearly assigned or have a more indirect influence on the signal pathway. Nevertheless, categorization revealed dysregulation of Wnt signaling genes that effects the entire pathway indicating extensive alterations compared to the healthy state. Especially, clustering of the major Wnt pathways initiators, such as Wnt10b, Wnt4, Wnt11 as well as Wnt16, Wnt5a, and Wnt2 appears to be striking. These Wnt ligands are highly upregulated in untreated and Wnt treated USH1C fibroblast when compared to their healthy counterparts (Figure 5A). A second cluster can be observed for several Wnt signaling related receptors and co-receptors of the plasma membrane, such as LRP5, FZD2, LRP6, PKD1, FZD4, and FZD1, which showed increased expression in untreated USH1C fibroblasts compared to healthy fibroblasts.

Overall, analysis of RNAseq data revealed aberrant transcriptomic patterns of several Wnt target and Wnt pathway genes in patient-derived USH1C fibroblasts, thereby strengthening *USH1C/*harmonin’s role for a functional Wnt signaling pathway.

### The translocation of *β*-catenin_K49 in human USH1C patient-derived cells

**FIGURE 6:**
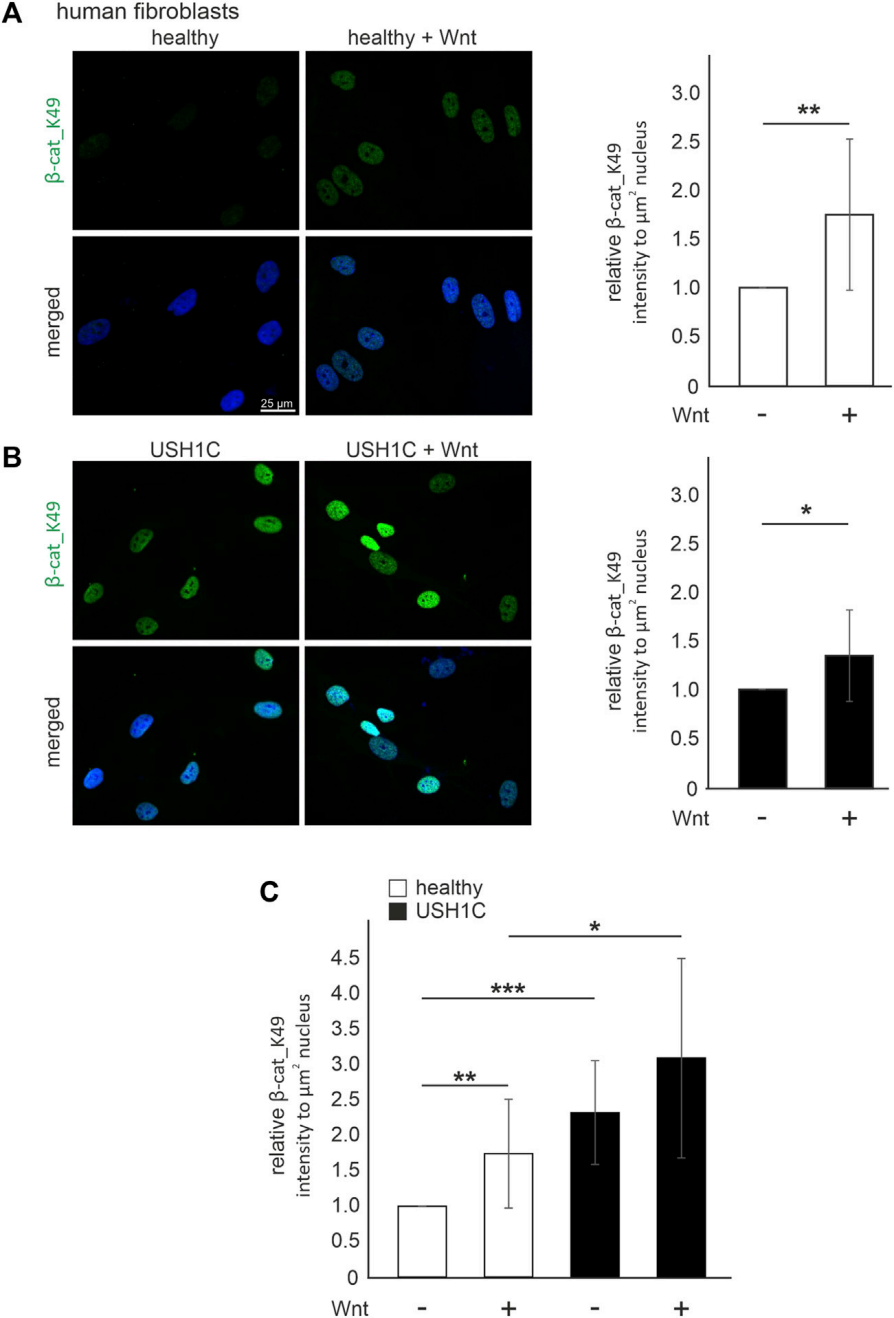
Harmonin leads to a decrease in Wnt signaling activity in patient-derived USH1C fibroblasts baering the patogenic *USH1C* ^R31*/R80Pfs*69^ mutations **(A)** Immunofluorescence analysis shows an increase of *β*-catenin_K49 (*β*-cat_K49) intensity (green) in Wnt stimulated human healthy fibroblasts. The nucleus is marked with DAPI (blue). Quantification reveals an increase of *β*-cat_K49 (1.74-fold) after stimulation with Wnt medium. Two-tailed Student’s *t*-test, **p ≤ 0.01; N = 12 experiments, cell number: Wnt- *n* = 1,219; Wnt+ *n* = 1,276. **(B)** Immunofluorescence analysis shows an increase of *β*-cat_K49 intensity (green) in Wnt stimulated *USH1C* ^R31*/R80Pfs*69^ patient derived fibroblasts. The nucleus is marked with DAPI (blue). Quantification reveals an increase of *β*-cat_lys49 (1.34-fold) after stimulation with Wnt medium. Two-tailed Student’s *t*-test, *p ≤ 0.05; N = 12 experiments, cell number: Wnt-, *n* = 1,282; Wnt+, *n* = 1,140. **(C)** Unstimulated *USH1C* ^R31*/R80Pfs*69^ fibroblasts show even higher levels of *β*-cat_K49 in stimulated healthy cells (unstimulated healthy = 1). Two-tailed Student’s *t*-test, *p ≤ 0.05, **p ≤ 0.01, ***p ≤ 0.001; N = 12 experiments.

To monitor cWnt signaling activation in human fibroblasts *in situ,* we stained acetylated *β*-catenin_K49 by immunocytochemistry in human healthy control fibroblasts and *USH1C* ^R31*/R80Pfs*69^ patient-derived fibroblasts. In healthy control fibroblasts and USH1C fibroblasts the intensities of the *β*-catenin_K49 immunofluorescence was significantly increased in the nuclei of fibroblasts cultured in Wnt conditioned medium (1.74-fold and 1.34-fold, respectively), when compared to fibroblasts cultured in control medium (Figures 6A, B). These comparisons indicated that the culturing in Wnt conditioned medium stimulated cWnt in both healthy and USH1C fibroblasts.

A comparison of the *β*-catenin_K49 immunofluorescence intensity between healthy and USH1C fibroblasts revealed that the intensities were significantly higher in USH1C fibroblasts under both conditions, unstimulated and stimulated conditions (Figure 6C). Taken together our data demonstrate that the activation of the cWnt signaling pathway is higher in cells lacking *USH1C*/harmonin suggesting *USH1C*/harmonin as negative regulator of in the cWnt-signaling pathway.

### Ataluren rescues the cWnt signaling phenotype in *USH1C-*deficient cells

**FIGURE 7:**
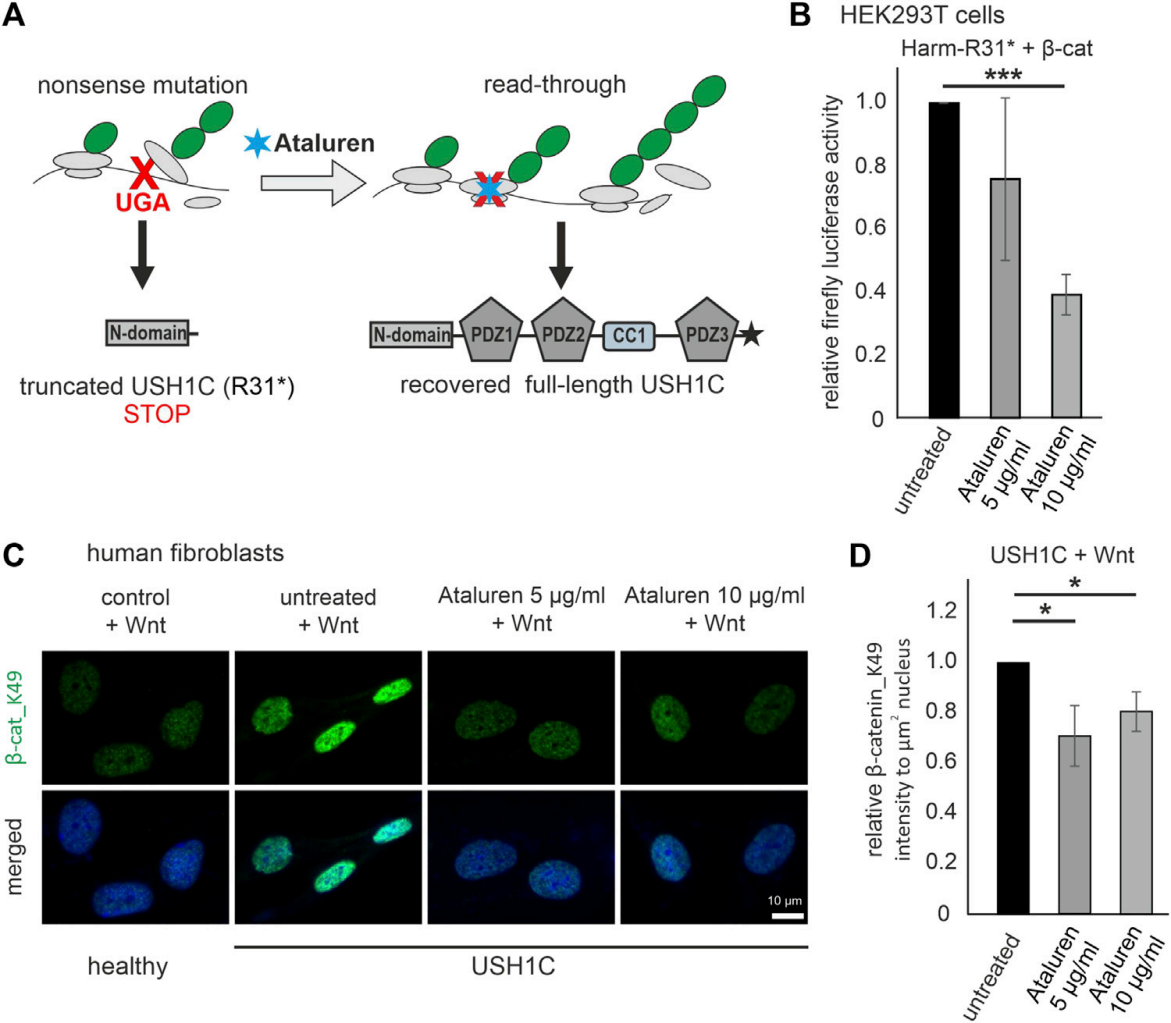
Treatment with Ataluren rescues cWnt in *USH1C*/harmonin-deficient cells. **(A)** Scheme of translational read-through of premature termination codons. Ataluren (blue asterisk) enhances translational read-through of the premature stop codon UGA (red) resulting in expression of full-length harmonin. **(B)** cWnt response in harmonin a1_R31*-GFP (Harm-R31*) and *β*-catenin (*β*-cat) transfected HEK293T cells measured by TCF/LEF luciferase activity. Treatment with 5 μg/mL and 10 μg/mL Ataluren shows significant reduction of cWnt response in Harm-R31* transfected cells. DMSO in all cells at 0.2%. Two-tailed Student’s *t*-tests, ***p ≤ 0.001; N = 3 experiments (three triplicates each condition). **(C)** Immunofluorescence analysis reveals significant decrease of *β*-cat_K49 (green) in Wnt stimulated *USH1C* (*USH1C* ^R31*/R80Pfs*69^) patient-derived fibroblasts after treatment with Ataluren (5, 10 μg/mL). The nucleus is marked with DAPI (blue). **(D)** Levels of *β*-cat_K49 were decreased by 0.3 (5 μg/mL) and 0.2 (10 μg/mL) in Ataluren treated cells. DMSO in all cells at 0.2%. Two-tailed Student’s *t*-tests, *p ≤ 0.05; N = 3 experiments, cell number: untreated, *n* = 246; Ataluren 5 μg/mL, *n* = 349 cells; 10 μg/mL, *n* = 277.

Next, we aimed to test whether the monitoring of the phenotype in cWnt signaling in cells can serve as a readout of therapeutic efficacy. The current hypothesis for the molecular mechanism of translational read-through is that in presence of TRIDs a near-cognate tRNA binds to the ribosomal A site and subsequently incorporates an amino acid into the nascent polypeptide at the position of the nonsense mutation (Figure 7A) (Roy et al., 2016). Consequently, the resulting restored proteins have insertion biases at the site of the nonsense mutation that might have an impact on the functionality of the protein and subsequently the therapeutic outcome. In our USH1C patient-derived cell line the p.R31* is due to a point mutation altering the triplet CGA, coding for arginine into the TGA stop mutation. In case of the present TGA nonsense mutation in *USH1C*, the amino acids leucine (L), serine (S), tryptophan (W), cysteine (C), glycine (G) and arginine (R) being the wild-type amino acid residue are predicted to be incorporated (Table 1). *In silico* tools, were used to predict whether the possible amino acid substitionsare expected to be tolerated. Of note, one missense variant c.92G>A p.Arg31Gln has been associated with USH1C-type USH (Zwaenepoel et al., 2001). However, *in silico* prediction programs are still erroneous in determining the specific effect of a likely pathogenic variant (Pater et al., 2019). Therefore, they should be interpreted with caution and should not be taken as a definitive approach (Matalonga et al., 2015; Samanta et al., 2019). Since our *in silico* analysis gave majoritarian damaging results, we extended our research and analysed the functionality of the recovered *USH1C*/harmonin protein.

**TABLE 1:** *In silico* missense variant assessment on potential disease-association of the predicted amino acid substitions resulting from translational read-through of *USH1C* c.91C>T; p.R31* nonsense mutation (i.e., wild-type codon -“-CGA-” to mutant premature termination codon “-TGA-”).

Codon position altered from PTC	Possible mispairing	Amino acid substitution	dbSNP	Polyphen-2 [0–1]	phyloP [−19.0; 10.9]	Grantham dist. [0–215]	Align GVGD [GV:353.86 - GD:0.00]	SIFT (score: 0. Median: 3.71)	MutationTaster (probability 1)	gnomAD MAF %
UGA	CGA	p.Arg31Arg (=wild-type)		—	—	—	—	—	—	—
	AGA									
	GGA	p.Arg31Gly	rs121908370	Probably damaging; 1	phyloP: 9.17 [−19.0, 11.0]	125 [0–215]	Class C0 (GV: 241.31—GD: 24.28)	DELETERIOUS (score: 0, median: 4.32)	Disease-causing; 1	0.00398
										10 het in 251248 alleles
UGA	UUA	p.Arg31Leu	—	Probably damaging; 1	—	102 [0–215]	Class C0 (GV: 241.31—GD: 90.63)	DELETERIOUS (score: 0, median: 4.32)	Disease-causing; 1	—
	UCA	p.Arg31Ser	—	Probably damaging; 1	—	110 [0–215]	Class C0 (GV: 241.31—GD: 21.04)	DELETERIOUS (score: 0, median: 4.32)	Disease-causing; 1	—
UGA	UGU	p.Arg31Cys		Probably damaging; 1		180 [0–215]	Class C0 (GV: 241.31—GD: 80.92)	DELETERIOUS (score: 0, median: 4.32)	Disease-causing; 1	—
	UGC									
	UGG	p.Arg31Trp		Probably damaging; 1	—	101 [0–215]	Class C0 (GV: 241.31—GD: 94.79)	DELETERIOUS (score: 0, median: 4.32)	Disease-causing; 1	—

We transfected HEK293T cells with Dual-Glo^®^ Luciferase cWnt reporters, GFP-tagged human harmonin a1 isoform with the R31* nonsense mutation (Harm-R31*), and with *β*-catenin for cWnt stimulation. After treatment with Ataluren (PTC124) (5 and 10 μg/mL in DMSO) the relative firefly luciferase activity was determined which demonstrates a doses-depended read-through efficacy of Ataluren: in the lower dose (5 μg/mL) Ataluren leads to a slight decrease in cWnt activity but the higher dose (10 μg/mL) significantly reduced cWnt signaling in Harm-R31* HEK293T cells when compared to DMSO treated control cells (Figure 7B).

Read-through efficacy strongly depends on the type of nonsense mutation as well as up- and downstream sequences and should be analysed on the patient specific background. Here, we analyzed the Ataluren´s translational read-through efficacy in human *USH1C* ^R31*/80Pfs*69^ patient-derived (USH1C) fibroblasts. We treated the fibroblasts stimulated in Wnt conditioned medium with Ataluren in two doses (5 μg/mL and 10 μg/mL) and subsequently monitored the nuclear localization of *β*-catenin_K49 by immunofluorescence microscopy (Figure 7C). We observed a significant reduction of *β*-catenin_K49 (∼20%–30%) in the nuclei of USH1C fibroblasts after treatment with Ataluren in both concentrations, 5 and 10 μg/mL in DMSO, compared to DMSO treated controls (Figure 7D).

In summary, our results show that hyperactivation of the cWnt pathway in the presence of Harm-R31* HEK293T cells or in skin fibroblasts from *USH1C* ^R31*/R80Pfs*69^ patients was corrected by administration of Ataluren. Thus, monitoring of cWnt signaling phenotype in cells may serve as an indicator of therapeutic efficacy in USH1C treatment.

## Discussion

**FIGURE 8:**
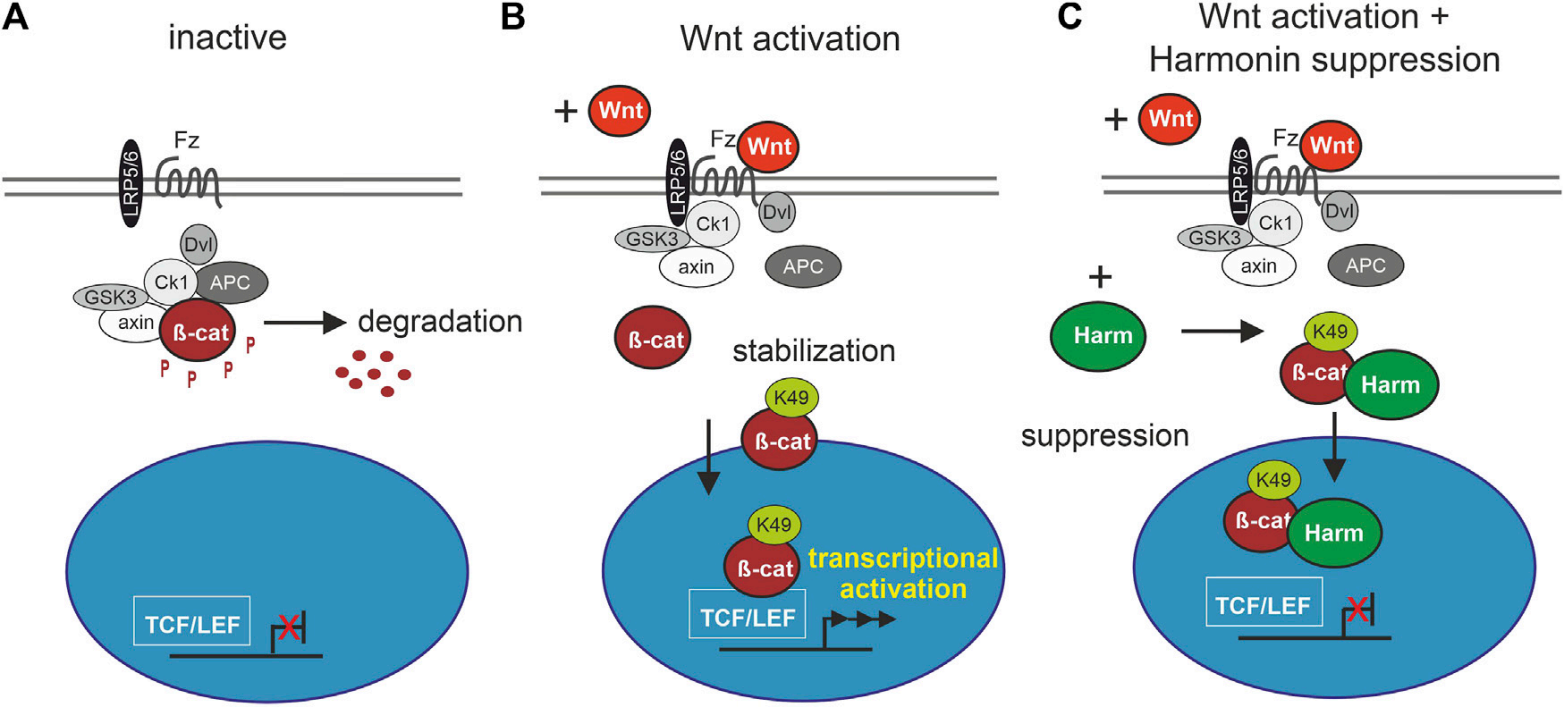
Schematic drawing of involvement of harmonin in cWnt signaling pathway. **(A)** In the inactive cWnt signaling pathway, absence of Wnt ligands leads to phosphorylation (P) of *β*-catenin (*β*-cat) by the destruction complex (grey and white circles) followed by protein degradation. **(B)** In presence of Wnt ligands, the pathway is activated by binding of Wnt to the receptor Frizzled (Fz), preventing the formation of the destruction complex. *β*-catenin can then be acetylated at K49 (*β*-cat_K49) resulting in its stabilization and nuclear translocation where it serves as transcriptional coactivator of TCF/LEF (T cell factor/lymphoid enhancing factor) Wnt target genes. **(C)** Harmonin (Harm) as negative regulator of cWnt signaling, suppresses the pathway by binding to *β*-cat_K49. The protein complex can still translocate to the nucleus but further transcription of cWnt target genes is blocked. Fz (Frizzled), LRP5/6 (lipoprotein receptor-related protein), Dvl (Dishevelled), APC (adenomatosis polyposis coli), axin, Ck1 (casein kinase 1α), GSK3 (glycogen synthase kinase 3).

cWnt signaling is highly regulated by several molecular network circles and its dysregulation can results in a multitude of diseases such as cancer or severe developmental defects (Ng et al., 2019). In particular, failure in downregulating cWnt signaling results in malfunctions highlighting the important role of suppressors of the pathway (Kumar and Tanwar, 2017). There are several modes of cWnt suppression: Suppression of cWnt signaling was found to be mediated by binding of secreted Wnt antagonists, such as Dickkopf-1 protein (DKK1) and sclerostin (SOST) to the LRP5/6 co-receptor (Mao et al., 2001; Delgado-Calle et al., 2017). Alternative mechanisms for the suppression of cWnt signaling in the cytoplasm were described in osteoblasts in which excessive activation of cWnt signaling downstream substrates is prevented by Schnurri-3 (SHN3), which attenuates ERK activity and thereby suppresses GSK3β (Delgado-Calle et al., 2017) and for the feedback suppressor axin which binds *β*-catenin for the recruitment to the destructor complex for proteasomal degradation (Fearon, 2011). Here, we show that the USH1C protein harmonin is also a potent regulator of cWnt signaling suppressing *β*-catenin transcriptional activity (Figure 8C).

The suppression of the cWnt signaling by harmonin is most probably achieved by direct binding of harmonin to *β*-catenin. We show this with the present interaction assays in mammalian cells and *in situ* by PLAs, confirming previous *in vitro* data (Johnston et al., 2004; Nagel-Wolfrum et al., 2022). The binary interaction of harmonin and *β*-catenin is mediated by binding of the C-terminal tail of *β*-catenin with its type I PBM (DTDL) (DuChez et al., 2019) to PDZ domains 1 and 3 on harmonin, with a slight preference for PDZ3 (Nagel-Wolfrum et al., 2022). Binding of the C-terminal PBM of *β*-catenin to PDZ domains has been previously described for interactions with other PDZ domain-containing proteins such as MAGI-1 and LIN7 (Dobrosotskaya and James, 2000; Perego et al., 2000). Such PDZ-mediated interactions are mostly related to the *β*-catenin’s function in cell-cell adhesions (Valenta et al., 2012), as we have recently shown for *β*-catenin-harmonin interaction in the human retina (Nagel-Wolfrum et al., 2022).

However, recent studies have further indicated that PBM-mediated interactions of *β*-catenin are also essential for its role as a coactivator in the cWnt pathway (DuChez et al., 2019). The interaction of the small PDZ protein EBP50 with the PBM of *β*-catenin potentiates its transcriptional activity in liver cancer cells (Shibata et al., 2003). In contrast, the binding of Tax-interacting Protein-1 (TIP-1) or Sorting Nexin 27 SNX27 *via* their PDZ domains with *β*-catenin’s PBM inhibits its transcriptional activity (Kanamori et al., 2003; DuChez et al., 2019). This is exactly what we also found for the harmonin-*β*-catenin interaction. Mechanistically, harmonin could influence the stability or subcellular localization of *β*-catenin or, alternatively, the formation of the TCF/*β*-catenin transcriptional activator complex or association of *β*-catenin with its transcriptional co-activators in the nucleus (Tago et al., 2000; Valenta et al., 2011; Valenta et al., 2012). Our data demonstrate that harmonin does not induce or increase the degradation nor alters the translocation of *β*-catenin into the nucleus as indicated by the results which we achieved by Western blots and cytochemistry, respectively (Figures 2B, 3A).

The ARM repeats R11–R12 and the C-terminal domain (CTD) of *β*-catenin recruits a multitude of transcriptional co-activators (Mosimann et al., 2009). In addition, conformational changes in the relative flexible CTD of *β*-catenin have been proposed to fold back on the central region affecting the binding of TCF and the formation of the TCF/β-catenin transcriptional activator complex (Castano et al., 2002; Solanas et al., 2004). Binding of harmonin may interfere with both, the binding of transcriptional co-activators and/or the fold back the CTD onto the central ARM repeats of *β*-catenin resulting in the repression of the *β*-catenin transcriptional activation.

Our present data consistently reveal that harmonin regulates cWnt signaling by the suppressing *β*-catenin transcriptional activity. In HEK293T cells, the overexpression of harmonin represses the transcriptional activity of *β*-catenin (Figure 3). We confirmed these findings in human primary dermal fibroblasts in which previously the activation of cWnt signaling by Wnt3a stimulation has been reported (Klapholz-Brown et al., 2007). The cWnt signaling was significantly increased in harmonin-deficient USH1C patient-derived fibroblasts (Figure 6), suggesting that WT harmonin also serves as a repressor of cWnt signaling in human fibroblasts. Such a suppressor function of harmonin is also reflected in the transcriptome of *USH1C*-deficient cells assessed by RNAseq analyses. The transcriptomes of both, Wnt stimulated and unstimulated USH1C cells are significantly different from the Wnt stimulated and unstimulated healthy controls (Supplementary Figure S2A; Figures 4, 5). *USH1C*/harmonin deficiency triggers extensive alterations in the transcriptome of cWnt signaling, consistent with defects found for the downregulation of other cWnt suppressors, such as the scaffold protein, axin (Wu et al., 2012). In particularly, the expression of numerous Wnt target genes is substantially altered in the absence of *USH1C*/harmonin further supporting an important role of *USH1C*/harmonin in the regulation of cWnt/*β*-catenin/TCF signaling gene expression program. In addition, we observed that harmonin deficiency has a direct impact on the expression of several genes enrolled in different levels of the cWnt signaling cascade and *β*-catenin/TCF transcriptional regulation, such as significant differential expression of genes for Wnt ligands, Wnt receptors and Wnt co-receptors as well as components of the *β*-catenin destruction complex and nuclear cWnt transcription factors and regulators. It will be interesting for future studies to decipher how these feedback loops are regulated.

Before designated as a gene causative of USH1C (Bitner-Glindzicz et al., 2000; Verpy et al., 2000), the *USH1C* gene product harmonin was first identified as an autoimmune antigen, synonymously named PDZ-73 and AIE-75, being upregulated in colorectal carcinomas and in cases of autoimmune enteropathy (AIE) (Kobayashi et al., 1999; Scanlan et al., 1999; Hirai et al., 2004). There is long standing evidence for an impact of cWnt signaling on cancer development especially for colorectal cancers (Klaus and Birchmeier, 2008). In colorectal cancers the cWnt signaling cascade is activated, e.g., by upregulation of Wnt ligands, and in turn cWnt pathway suppressors, such as axin2, are upregulated (Fearon, 2011; Wu et al., 2012; Nie et al., 2020), which is coincident with the present and previous findings for *USH1C*/harmonin (Kobayashi et al., 1999). Our results show that harmonin has a similar function to axin2 in the canonical cWnt signaling cascade in the normal cell under physiological conditions and acts as a negative regulator or suppressor of cWnt signaling (Wu et al., 2012). Accordingly, like axin2, *USH1C*/harmonin may be a potential target for colorectal cancer therapy (Chen et al., 2009; Novellasdemunt et al., 2015).

Numerous pathologic variants (mutations) in *USH1C* gene are causative for Usher syndrome type 1 (https://databases.lovd.nl/shared/genes/USH1C), characterized by congenital profound deafness and vestibular dysfunction, combined with visual loss before puberty (Fuster-Garcia et al., 2021; Delmaghani and El-Amraoui, 2022), affecting the sensory epithelia in both the inner ear and the eye. In the inner ear, both the non-canonical Wnt (PCP) and the cWnt signaling pathway are important, first during the early development and later during the differentiation of the mechanosensitive hair cells (Jacques et al., 2012). The USH1C protein harmonin is essential for the correct differentiation of the stereocilia bundles of hair cells (Boeda et al., 2002; Johnson et al., 2003). In its absence the arrangement of the hair bundles is disturbed, a phenotype which can be also found in deregulated cWnt signaling (Jacques et al., 2012; Deng et al., 2021). However, present GO term analysis categorized differentially expressed genes in USH1C-deficient fibroblasts to “early neural differentiation” and “early inner ear/cochlear development” (Figure 4B), which points to an additional role of harmonin during early development in particular in the inner ear.

Dynamic activation of the cWnt signaling pathway has been described throughout retinal development, suggesting a role in multiple aspects of retinal development and homeostasis (Liu et al., 2006). Considering the late onset of the retinal degeneration in USH1 we do not expect the regulation of the cWnt signaling by *USH1C*/harmonin to play a role during retinal development. In the adult mammalian retina cWnt signaling regulates the proliferation and neurogenic potential of Müller glial cells (Angbohang et al., 2016; Yao et al., 2016). Given its high expression in the Müller glia cells of the human retina (Cowan et al., 2020; Nagel-Wolfrum et al., 2022), harmonin may also serve as a suppressor of cWnt signaling and proliferation of the Müller glial cells in the retina. A regulatory role of harmonin in Müller glial cells is supported by data of our recent study revealing increased expression of glial fibrillary acid protein (GFAP) in the *USH1C*/harmonin deficient retina of a humanized *USH1C* porcine model (Grotz et al., 2022).

We have previously shown that patient-derived dermal fibroblasts are suitable to test potential treatment options for human hereditary retinal disorders (Samanta et al., 2019; Grotz et al., 2022; Nagel-Wolfrum et al., 2022). A query and comparison of variants listed in the disease-associated mutation database HGMDpro and variant database gnomAD (https://gnomad.broadinstitute.org/gene/ENSG00000006611) identified 87 disease-associated variants and only 22 missense mutations (25%). In contrast, gnomAD lists 2,105 variants, of which 978 are exonic and 637 are accounted by missense variants (637/978, 65%). This might support the notion, that disease-associated variants in *USH1C* are mostly deleterious if they are null alleles (i.e., nonsense, frame-shift, splicing), while *USH1C* is tolerant to missense mutations. This may indicate that *USH1C*/harmonin protein tolerates amino acid substitutions, and thus read-through is a very promising approach for patients with a nonsense variant. Present results further highlight the great potential of translational read-through therapy for defects caused by nonsense mutations confirming our previous data [summarized in (Nagel-Wolfrum et al., 2016)]. Moreover, the cellular disease models have also great potential to screen and test candidate drugs for treatments associated with altered cWnt activity, including colorectal cancer.

## Conclusion

In conclusion, we provide several lines of evidence that the USH1C protein harmonin is a potent suppressor of cWnt pathway by interacting with the cWnt pathway coactivator *β*-catenin. We demonstrate that *USH1C*/harmonin deficiency in *USH1C* patient-derived fibroblasts significantly alters not only the expression of Wnt target genes but also of genes related to cWnt signaling pathway. Finally, we proof that the transitional read-through drug Ataluren restores the cWnt phenotype in HEK293T cells and patient-derived fibroblasts bearing an early nonsense mutation in *USH1C*. The novel role of *USH1C*/harmonin in the control of cWnt signaling identified here opens new avenues of research to unravel the pathomechanism of USH, identify new therapeutic targets, and evaluate treatment options in patient-derived cell models.

## Data Availability

The data presented in this study have been deposited in the NCBI Sequence Read Archive (SRA). It is accessible under the BioProject accession number PRJNA925096 or with this link: https://www.ncbi.nlm.nih.gov/sra/PRJNA925096.

## Glossary

Ac: acetate
AIE: autoimmune enteropathy
AJ: adherent junctions
APC: adenomatosis polyposis coli
CBY1: chibby
CK1: casein kinase 1
CTD: C-terminal domain
cWnt: canonical Wnt signaling
DAPI: 4′,6-diamidino-2-phenylindole
DEG: differential expressed gene
DKK: Dickkopf
DMD: Duchenne muscular dystrophy
DMEM: Dulbecco’s modified Eagle’s medium
DTDL: PDZ binding motif of *β*-catenin
Dvl: dishevelled
FBS: fetal bovine serum
FDR: false discovery rate
FPKM: fragments per kilobase per million mapped fragments
Fz: frizzled
GFAP: glial fibrillary acid protein
GFP: green fluorescent protein
GO: gene ontology
GSK3: glycogen synthase kinase 3
h: hour
Harm: harmonin
HEK: human embryonic kidney
LRP5/6: lipoprotein receptor-related protein
PBM: PDZ binding motif
PBS: phosphate-buffered saline
PCA: principal component analysis
PDZ: PSD-95, DLG, and ZO-1
PFA: paraformaldehyde
PLA: proximity ligation assay
PolyPhen-2: polymorphism phenotyping v2
PP2: protein phosphatase
ROI: region of interest
RP: retinitis pigmentosa
SHN3: Schnurri 3
SOST: sclerostin
SIFT: sorting intolerant from tolerant
TCF/LEF: T cell factor/lymphoid enhancing factor
TRID: translational read-through inducing drug
USH: Usher syndrome
*USH1C* fibroblasts: dermal fibroblasts derived from skin biopsies of *USH1C* patient with confirmed *USH1C* ^R31*/80Pfs*69^ mutations
WB: Western blot
WMS: Wnt medium stimulation of Wnt signaling pathway
*β*-cat_K49: *β*-catenin acetylated at lysin residue K49
*β*-catS: stimulation of Wnt signaling pathway by overexpression of *β*-catenin
*β*-cat: *β*-catenin
