# Supplementary material for: The Usher syndrome 1C protein harmonin regulates canonical Wnt signaling
Source: Front Cell Dev Biol. 2023 Feb 8;11:1130058. doi: 10.3389/fcell.2023.1130058 (PMC9944737; doi:10.3389/fcell.2023.1130058)
Supplement: Supplementary file 7 [file DataSheet1.docx]

Supplementary Material

The Usher syndrome 1C protein harmonin regulates canonical Wnt signaling

Jessica Schäfer^1^, Nicole Wenck^1^, Katharina Janik^1^, Joshua Linnert^1^, Katarina Stingl^2^, Susanne Kohl^3^, Kerstin Nagel-Wolfrum^1,4^, Uwe Wolfrum^1*^

^1^Institute of Molecular Physiology, Molecular Cell Biology and Photoreceptor Cell Biology, Johannes Gutenberg University Mainz, Germany; ^2^ University Eye Hospital, Centre for Ophthalmology, University of Tubingen, Germany; ^3^Institute for Ophthalmic Research, Centre for Ophthalmology, University of Tubingen, Germany; ^4^Institute of Developmental Biology and Neurobiology, Johannes Gutenberg University Mainz, Germany

*Corresponding Author: Prof. Dr. Uwe Wolfrum, Institute of Molecular Physiology, Molecular Biology, Johannes Gutenberg University Mainz, Hanns-Dieter-Hüsch-Weg 17, 55128 Mainz, Germany. Phone: + 49-6131-39-25148; e-mail: wolfrum@uni-mainz.de

# Supplementary Data

# Supplementary Figures and Tables

For more information on Supplementary Material and for details on the different file types accepted, please see [here](https://www.frontiersin.org/guidelines/author-guidelines#supplementary-material).

## Supplementary Figures


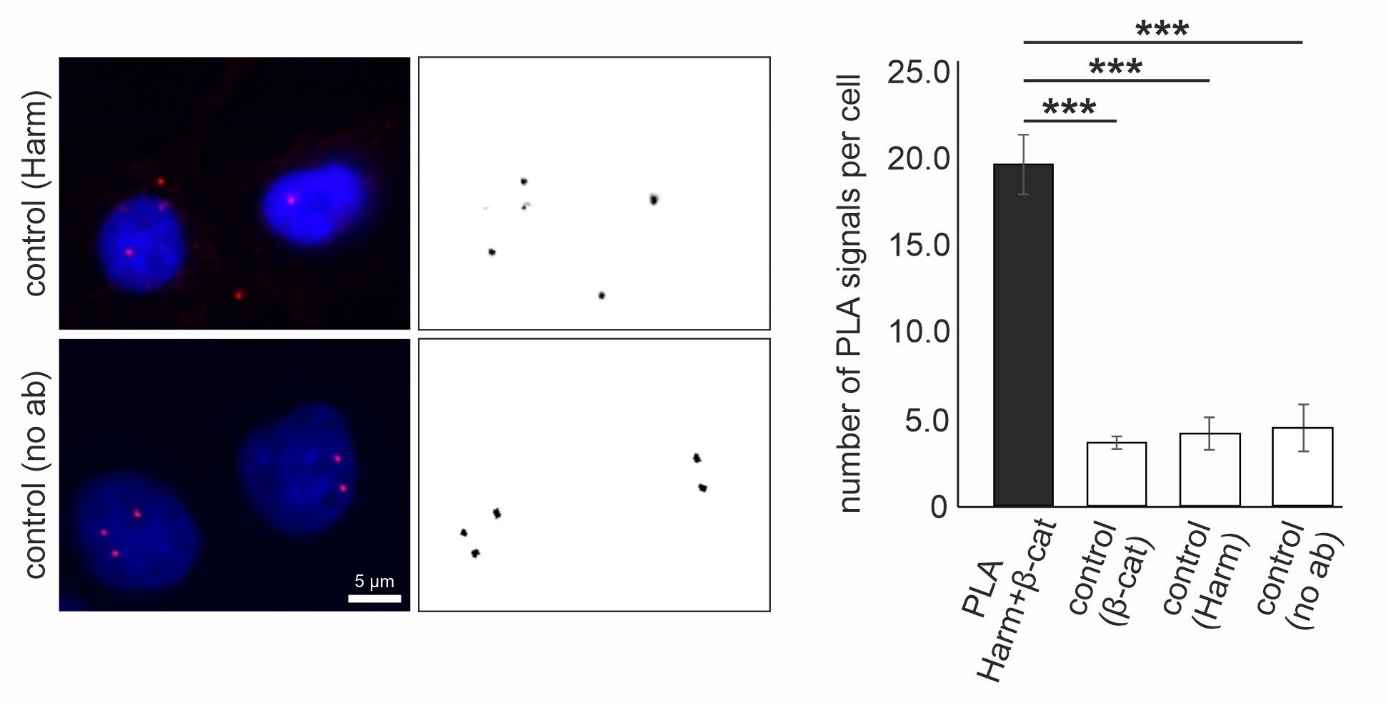


**Supplemental Fig. S1. Proximity ligation assay staining for controls**

Representative proximity ligation assay (PLA) control stainings (red, left) in HEK293T cells. Negative controls were probed with anti-β-catenin (β-cat), anti-harmonin (Harm) or without primary antibodies (no ab) and paired with the rabbit- and mouse-IgG-specific oligonucleotide-labelled antibodies. Cell nuclei are marked with DAPI (blue). Statistical analysis reveals an increase in number of signals of 3-fold in the PLA compared to all three controls. Two-tailed Student’s t-test, ***p≤0.001; N=3 experiments.


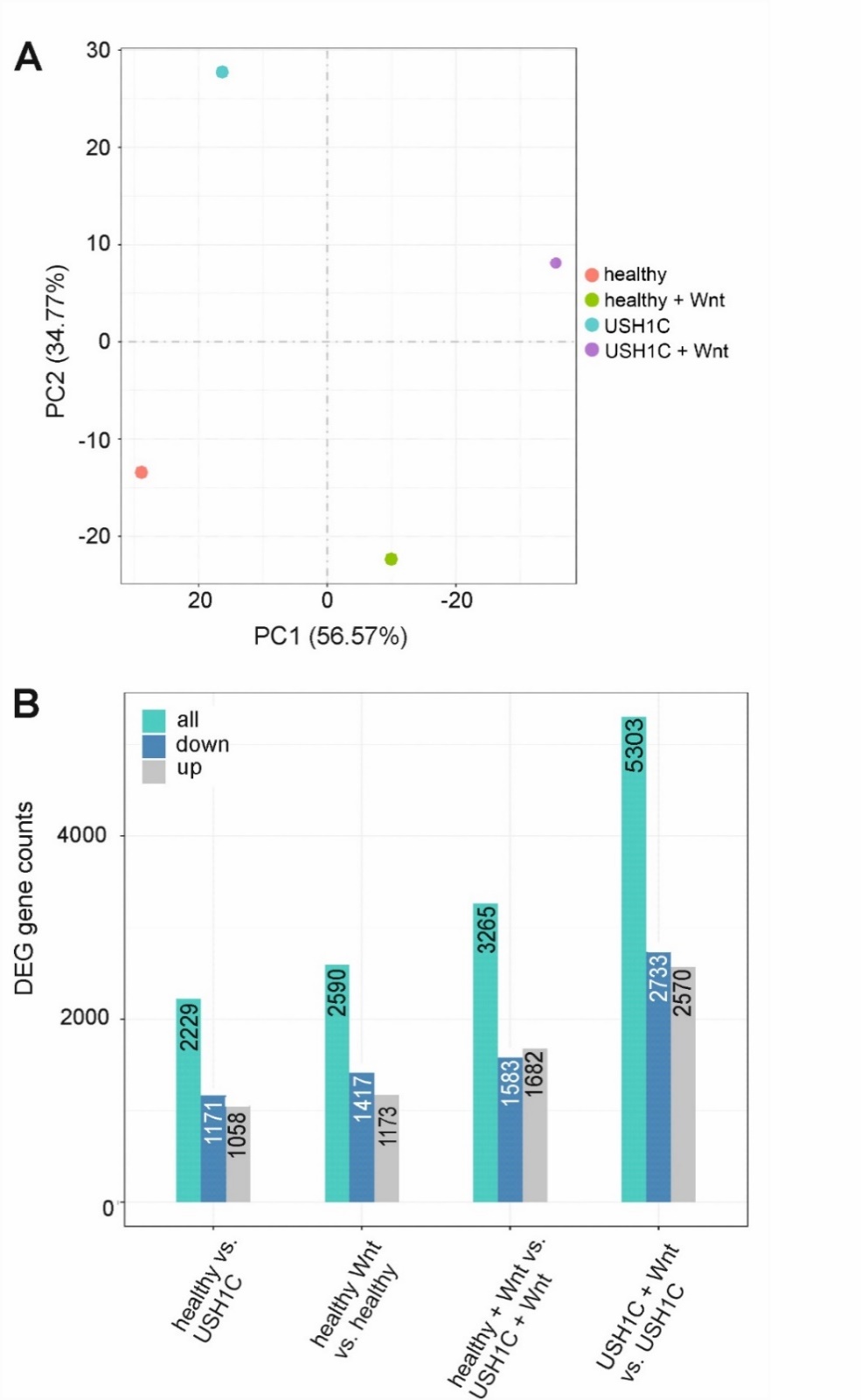


**Supplemental Fig. S2. RNAseq analysis of human healthy and patient-derived USH1C^R31*/R80Pfs*69^ (USH1C) fibroblasts.**

(**A**) Principle Component Analysis (PCA) shows the differences of transcriptomes between untreated (healthy (orange), USH1C^R31*/R80Pfs*69^ (blue)) and Wnt treated fibroblasts (healthy + Wnt (green), USH1C^R31*/R80Pfs*69^ + Wnt (purple)) with each other. (**B**) Counts of differentially expressed genes (DEG) when comparing the distinct treated human fibroblasts. Green bars indicate the total counts of differentially expressed genes, grey bars indicate an upregulation and blue bars a downregulation of gene expression.

**Supplemental Tables:**

Supplemental Table S1. FPKM-values of DEGs in unstimulated and WMS healthy and USH1C patient-derived fibroblasts, which were used for heatmapping.

Supplemental Table S2. FPKM-values of DEGs in unstimulated and WMS USH1C patient-derived fibroblasts.

Supplemental Table S3. FPKM-values of DEGs in unstimulated and WMS healthy fibroblasts.

Supplemental Table S4. FPKM-values of DEGs in WMS healthy and USH1C patient-derived fibroblasts.

Supplemental Table S5. FPKM-values of DEGs in unstimulated healthy and USH1C patient-derived fibroblasts.

Supplemental Table S6. Results of GO enrichment analysis of biological processes for 40 dysregulated Wnt target genes in unstimulated and WMS healthy and USH1C patient-derived fibroblasts.
